# Radiotherapy‐Induced Side Effects: Epidemiology, Pathogenesis, Prevention and Treatments

**DOI:** 10.1002/mco2.71034

**Published:** 2026-09-27

**Authors:** Xiaowen Ma, Xiaoxuan Zhang, Pujun Zhao, Jiaxin Yang, Zhihao Li, Xiaoyang Han, Yixuan Wang, Zhenzhen Cui, Nian Liu, Min Li

**Affiliations:** ^1^ Department of Pharmacy The 960th Hospital of PLA Jinan China; ^2^ Medical Support Center The 960th Hospital of PLA Jinan China; ^3^ Department of Nuclear Medicine The 960th Hospital of PLA Jinan China; ^4^ Department of Pharmacy Shandong University of Traditional Chinese Medicine Jinan China; ^5^ Department of Pharmacy Shandong Second Medical University Weifang China; ^6^ Department of Pharmacy Jinzhou Medical University Jinzhou China

**Keywords:** epidemiology, pathogenesis, prevention, radiation‐induced injury, risk factors, treatment

## Abstract

The widespread application of radiotherapy for malignancies underscores the urgent clinical challenge of radiation‐induced multiorgan injury, which severely impacts patient prognosis and quality of life. The heart, skin, lungs, intestines, hematopoietic system, oral, kidney, and brain are the most frequently affected sites, each following distinct and complex injury trajectories. Despite growing research into these organ‐specific injuries, a substantial translational gap remains between mechanistic insights and effective therapeutic interventions. This review systematically delineates the unique epidemiological and pathological processes in these eight critical systems. It then critically evaluates current clinical management alongside emerging therapeutic strategies, including advanced radioprotectors, mitigators, antifibrotic agents, and innovative stem cell therapies. Central to our analysis is the synthesis of these findings into a comprehensive view of the complex interactive networks and functional imbalances driven by aberrant intercellular communication within damaged tissues. By offering a systematic analysis of mechanism‐network‐targeted therapeutics, this review establishes a critical framework to guide the future development of more effective, integrated, and personalized patient care strategies in radiation oncology.

## Graphical abstract

Radiotherapy for malignancies frequently injures the organs’ systems through oxidative stress, fibrosis, inflammation, and vascular damage. This review systematically maps the epidemiology, pathogenesis, and emerging therapies to propose a mechanism‑network‑targeted framework for integrated, personalized care in radiation oncology.

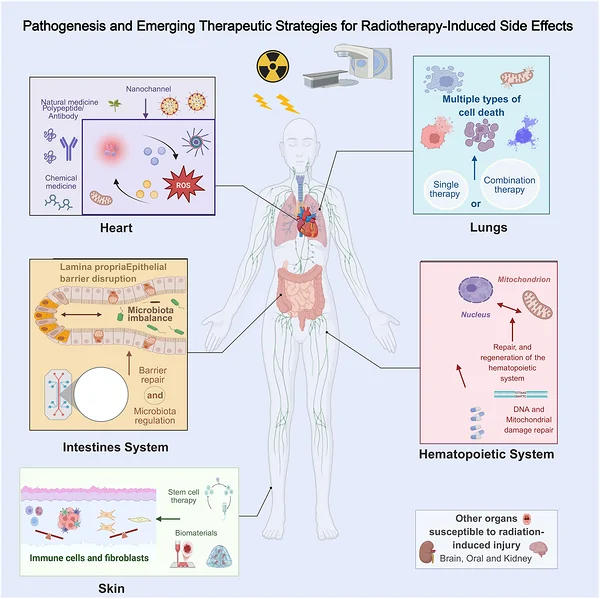

## 1 Introduction

Radiotherapy serves as a cornerstone in the management of these malignancies, functioning both as a curative modality—often combined with chemotherapy—for primary tumors [1], and as a palliative approach for advanced or metastatic disease to alleviate symptoms and prolong survival [2]. More than 50% of patients with malignancies receive radiotherapy during their treatment course [3]. Despite its indispensable role, radiotherapy inevitably causes adverse effects in normal tissues—including the heart, skin, lungs, intestines, hematopoietic system—posing a major challenge in contemporary radiation oncology. Radiation‐induced injury arises when exposure exceeds the threshold dose for biological effects, leading to pathophysiological damage and stromal changes in normal tissues [4, 5]. Since the discovery of X‐rays, medical exposure, primarily through radiotherapy, has become a leading cause of such injury [6]. The past two decades have witnessed transformative advances in radiation therapy. Precision techniques, such as, three‐dimensional conformal radiotherapy, intensity‐modulated radiotherapy, volumetric modulated arc therapy, stereotactic body radiotherapy, and proton therapy enable precise tumor targeting while sparing surrounding healthy tissues [2]. Proton therapy, for instance, has demonstrated reduced cardiac, hematologic, and pulmonary toxicity compared with conventional photon radiotherapy [7]. Carbon ion radiotherapy offers superior biological efficacy owing to its higher linear energy transfer [8] while FLASH radiotherapy, delivered at ultra‐high dose rates (>40 Gy/s), shows promise in reducing radiation‐induced normal tissue toxicity without compromising tumor control [9, 10]. Additionally, optimized treatment protocols—through beam‐angles adjustment and precise dose distribution—further reduce normal‐tissue exposure and injury risk [11]. Radiomics combined with artificial intelligence (AI) have enhanced precision further: quantitative imaging features extracted from pretreatment CT scan can feed predictive models of individual radiation‐injury risk, and AI‐based optimization of radiotherapy parameters aids personalized treatment planning, improving accuracy while lowering the risk of harm [12, 13].

Radioimmunotherapy represents another innovative strategy, integrating the local cytotoxic effects of radiation with systemic antitumor immunity. Immune checkpoint inhibitors, such as, PD‐1/PD‐L1 inhibitors and CTLA‐4 inhibitors, synergistically amplify radiotherapy‐induced immune responses by relieving immunosuppressive signals. Landmark clinical trials, including PACIFIC and ADRIATIC, have shown that consolidation immunotherapy after chemo‐radiotherapy improves progression‐free and overall survival in patients with locally advanced non‐small cell lung cancer (NSCLC) and limited‐stage small cell lung cancer [14, 15, 16, 17]. Recent nanotechnology‑based strategies further potentiate this synergy; for instance, nanointegrated approaches that exploit tumor lipid metabolism can induce ferroptosis‐mediated immunogenic cell death under low‑dose radiation, reducing normal‑tissue injury risk while enhancing antitumor immunity through the release of damage‑associated molecular patterns and tumor‑associated antigens [18]. These innovative nano‑radioimmunotherapy strategies have demonstrated superior tumor control and acceptable safety profiles in animal models [19]. Despite these advances, radiation‑induced toxicities remain an unavoidable challenge, underscoring the need for continued research into protective strategies and optimized treatment protocols [20].

While current clinical therapies can partially mitigate radiation‐induced organ damage, their effectiveness remains limited [7]. For radiation‐induced lung disease (RILI), conventional treatments—including nitric oxide (NO) [21], antioxidants [22], pulmonary surfactants [23], glucocorticoids [24], immunomodulators [25], steroids [26], and granulocyte–macrophage colony‐stimulating factor (GM‐CSF) [27]—primarily target inflammatory pathways. However, these approaches are constrained by suboptimal efficacy, prolonged treatment requirements, and significant side‐effects [28]. Notably, many radioprotective agents that are effective in rodent models require administration either before or immediately after radiation exposure, limiting their clinical utility [7]. In radiation‐induced cardiac disease (RICD), repurposed cardiovascular medications like statins and ACE inhibitors confer some benefits [2], but no therapies specifically address chronic radiation‐induced myocardial fibrosis. Moreover, no approved secondary prevention strategies exist to reduce cardiovascular risk in cancer survivors following radiotherapy [29, 30]. For radiation dermatitis, the International Delphi Consensus recommends preventive measures including photobiomodulation therapy and topical applications (Mepitel film, hydrofilm, corticosteroids, and olive oil) [31]; however, these interventions show variable site‑dependent efficacy and may cause adverse effects [32]. Early diagnostic uncertainty further compromises treatment outcomes. The maximum cardiac radiation dose, concurrent cardiotoxic systemic therapies, prior treatment history, and baseline cardiovascular risk factors all contribute to radiation‑induced cardiac injury, and the lack of validated biomarkers for RICD risk stratification hampers early and precise diagnosis. Across all organs, there is a pressing need to develop and identify biomarkers, parameters, and predictive models that can effectively stratify injury risk [29, 33, 34]. Existing diagnostic and therapeutic approaches for radiation injury typically target single mechanisms while overlooking synergistic interactions, resulting in limited efficacy or significant side effects. Most related research remains in the preclinical stage, with only a handful of ongoing clinical trials, indicating that clinical translation requires further investigation [28]. A thorough understanding of the complex molecular network mechanisms underlying organ‑specific radiation injuries is therefore essential for developing effective diagnostic and protective strategies.

**FIGURE 1:**
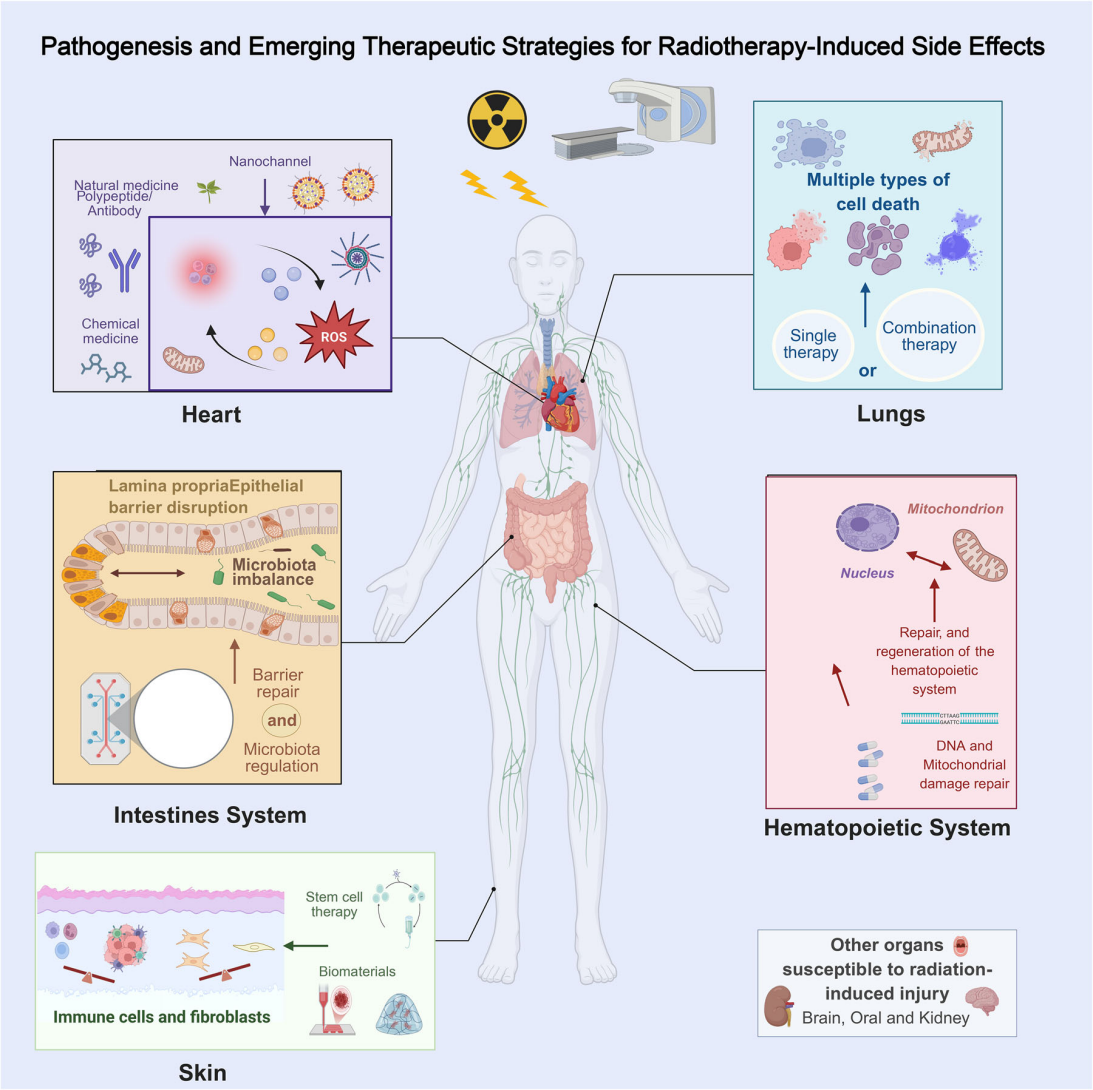
Pathogenesis and emerging therapeutic strategies for radiotherapy‐induced side effects. This figure summarizes radiation‐induced toxicities in the heart (redox–inflammation crosstalk), lungs (convergent cell death pathways), skin (fibroblast and immune cell roles), hematopoietic system (mitochondrial and DNA damage), intestine (epithelial barrier–flora dysbiosis), and other organs susceptible to radiation‐induced injury. Emerging therapies—including nanomaterials and stem cells—are highlighted, along with a comparison of combination versus single‐target approaches. Abbreviations: RNS: reactive nitrogen species; FGF: fibroblast growth factor; cGMP: cyclic guanosine monophosphate; IKKβ: IκB kinase beta.

This review provides a comprehensive examination of the pathological mechanisms of radiotherapy‐induced injury, focusing on the hematological, dermatological, cardiac, intestinal, pulmonary, oral, renal, and brain systems. By systematically elucidating the intricate interaction networks among molecular pathways, we aim to bridge current knowledge gaps in radiation biology. We critically evaluate existing and emerging pharmaceutical interventions for multiorgan radiation injury, offering insights into their therapeutic potential and limitations (Figure 1). Integrating cutting‑edge research, this work establishes a framework for each organ system, covering (1) epidemiology, (2) pathological features, and (3) management of radiation‑induced injury. Finally, we identify key challenges and propose future research directions to advance the field of radiation injury management.

## 2 Organ‐at‐Risk Injuries in Radiotherapy: Dose, Time, and Individual Susceptibility

Excessive radiotherapy inevitably affects multiple tissues and organs, such as, the lungs, hematopoietic tissue and bone marrow (BM), heart and its substructures, esophagus, and thoracic integument [7]. Among these, the skin—as the body's outermost barrier—invariably undergoes radiation exposure regardless of the delivery technique, with radiation‐induced dermatitis being the most frequent radiotherapy‐related complication, particularly for superficially located tumors, significantly impairing patients’ quality of life [35]. The BM and hematopoietic system, owing to their high proliferative activity, are exquisitely radiosensitive, presenting a major challenge in treatment planning [36]. The heart, as a critical organ at risk during radiotherapy, comprises multiple structural components including muscle, vasculature, valves, and the conduction system. These distinct structures possess different radiation tolerance doses and exhibit interdependent biological functions; damage to any structure may precipitate long‐term cardiovascular disease [37, 38]. Pulmonary tissue contains abundant alveolar epithelial cells and vascular endothelial cells, which are highly sensitive to radiation‐induced DNA damage and apoptosis [39], whilst continuous gas exchange maintains the lung in a state of high oxygenation [40]. Collectively, the anatomical and biological features render the heart, lungs, blood, and skin the primary organs at risk during radiation [41].

Clinical evidence consistently demonstrates the dose‐dependent nature of such injuries. In breast cancer and lymphoma, a linear dose–response relationship is observed for cardiotoxicity, with each 1 Gy increase in mean heart dose elevating the relative risk of major cardiac events by 4–17% [42, 43]. Lung cancer patients (typically receiving higher cardiac doses) experience a >10% incidence of severe (Grade ≥3) cardiac toxicity, which correlates strongly with thoracic radiation dose to thoracic bones [44]. Hematopoietic acute radiation syndrome (H‐ARS) manifests at 2–6 Gy (whole‐body exposure) and demonstrates clear dose‐dependence across the 2–10 Gy range [45]. Lung injury risk escalates with mean lung dose: <10 Gy associates with a 10% RILI incidence, whereas 11–20 Gy raises this to 16% [46, 47]. The spatial interplay between organs at risk and target volumes further complicates dose distribution [41]. For instance, cardiac dose reduction strategies in photon therapy may inadvertently increase pulmonary exposure due to the heart–lung geometric coupling [37, 38]. Furthermore, clinical experience with different fractionation regimens provides corroborative evidence of dose dependency. Retrospective studies indicate that hypofractionated radiotherapy in the context of concurrent chemotherapy is associated with reduced risk of severe lymphopenia and improved clinical outcomes in patients with locally advanced NSCLC [48, 49]. Ultra‐hypofractionation (26 Gy in five fractions) has proven noninferior to moderate hypofractionation, and in the Fast‐Forward trial, constraints limiting the volume receiving 7 Gy to <5% and the volume receiving 1.5 Gy to <30% demonstrated low rates of late cardiac toxicity [50]. Therefore, balancing the complex dose‐dependent relationship between radiotherapy and radiation‐induced injury has consistently posed a formidable challenge in the formulation of clinical radiotherapy protocols.

The temporal characteristics of radiotherapy exert equally important clinical implications on radiation‐induced injury [51]. The clinical manifestations of radiation‐induced injury typically occur from several weeks to several decades following radiotherapy, influenced by radiation dose and anatomical targeting. For instance, RIHD is characterized by a prolonged latency period (commonly 5–10 years postexposure) [52], with risk appearing to emerge several years after radiation exposure and persisting for two to three decades. Notably, more than 50% of ischemic events occur beyond 10 years following initial radiation exposure [53]. Additionally, individual factors like age and baseline comorbidities markedly influence toxicity, with older patients exhibiting poorer radiotherapy tolerance [54, 55, 56]. Other pertinent factors including age, sex, smoking history, comorbidities, and tumor type all present obstacles to clinical radiotherapy dose escalation [57].

Given their high incidence, pronounced dose‐dependence, and substantial interpatient variability, injuries to these organ systems represent major barriers to dose escalation, diminish quality of life, and contribute to noncancer mortality, underscoring the need for investigating the injury mechanisms of radiotherapy, refined radiation techniques, and personalized risk mitigation strategies [58, 59]. These five systems were selected because they collectively represent the major clinically dose‐limiting toxicities of radiotherapy: cardiac and pulmonary injury contribute substantially to noncancer morbidity and mortality, skin injury is among the most frequent and visible toxicities, hematological injury limits systemic treatment tolerance, and intestinal injury is a major determinant of abdominal and pelvic radiotherapy‐related morbidity. Although other organs can also be affected by radiotherapy, the heart, lungs, skin, hematopoietic system, and intestines cover critical vascular, parenchymal, barrier, immune‐regenerative, and mucosal tissue contexts. Given the shared mechanistic basis of radiation‐induced tissue injury, our discussion of other commonly affected organs—including the kidney, oral cavity, and brain—focuses primarily on disease progression and therapeutic strategies. Taken together, the following organ‐specific sections consequently adopt a consistent sequence of epidemiology, pathogenesis, prevention, and treatment to connect clinical burden with mechanistic interpretation and actionable intervention strategies.

## 3 Radiation‐Induced Cardiac Disease

Because anatomical substructures—including the coronary arteries, myocardium, pericardium, cardiac valves, and conduction system—exhibit distinct responses to radiation, RICD is characterized by a prolonged latency and highly heterogeneous clinical manifestations [60, 61]. Radiation‐induced oxidative stress and inflammation form a self‐reinforcing pathological circuit that not only constitutes a shared mechanism of injury across cardiac substructures but also drives the transition from early tissue damage to late cardiac remodeling. Against this anatomical and mechanistic complexity, the therapeutic modality and delivery capacity of pharmacological interventions determine which pathological targets can be effectively engaged and, ultimately, their efficacy in mitigating RICD.

### 3.1 Epidemiology of RICD

RICD represents the most prevalent iatrogenic complication of radiotherapy, particularly affecting patients with left‐sided breast cancer, Hodgkin's lymphoma, and mediastinal tumors [60, 62]. Epidemiological evidence reveals a clear dose–response relationship, with each 2–4 Gy increase in the mean heart dose elevating the relative risk of major cardiovascular events by 4–16% [43, 61]. Notably, breast cancer patients receiving radiotherapy exhibit a 1.38‐fold higher cardiac mortality risk (95% CI 1.18–1.62, *p* < 0.001) compared with nonirradiated controls [63], establishing RICD as a critical determinant of long‐term survival in cancer survivors. The clinical spectrum of RICD encompasses diverse pathologies including coronary artery disease, heart failure, cardiomyopathy, pericardial disease, conduction system abnormalities, and valvular heart disease. These manifestations typically exhibit delayed onset and significant heterogeneity [64], with many patients presenting with multiple concurrent cardiac complications at diagnosis. This complexity underscores the urgent need for improved early prediction methods. Emerging researches highlight multifactorial pathogenesis, where radiotherapy combined with immunotherapy has been shown to elevate cardiac IgG levels in murine models, potentiating toxicity [65]. Additional risk modifiers include immune checkpoint inhibitors, age at exposure, metabolic conditions (diabetes, hypertension), smoking, and prior cytotoxic chemotherapy [66]. The pathophysiological overlap between RICD and age‐related cardiovascular diseases further complicates clinical differentiation, leading to diagnostic challenges and elevated mortality [64]. Therefore, a thorough understanding of the pathophysiological mechanisms underlying RICD is essential for developing targeted prevention and therapeutic strategies.

### 3.2 Pathogenesis of RICD

**FIGURE 2:**
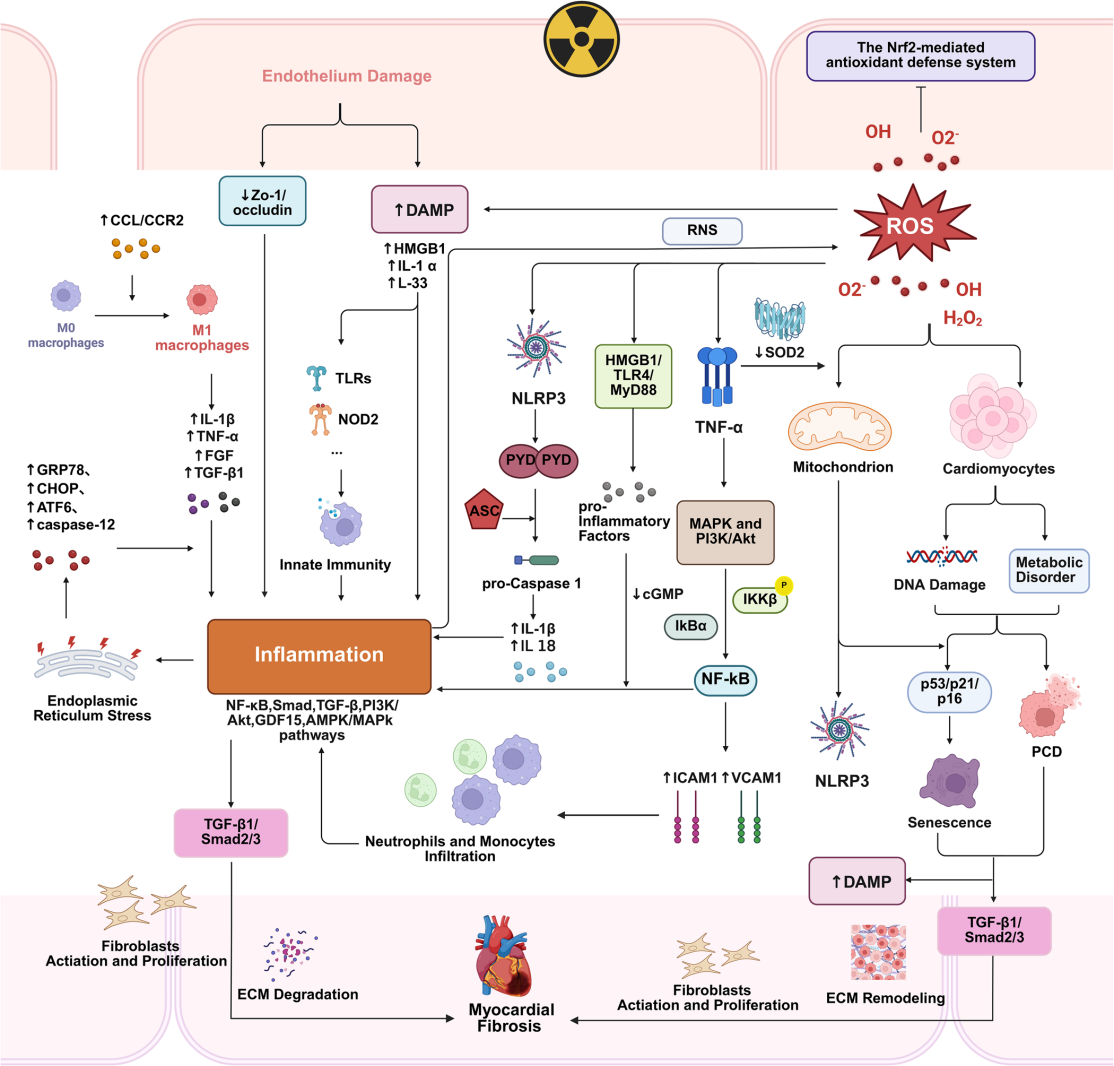
Core pathological mechanism of radiation‐induced heart disease: the inflammation–oxidative stress cascade reaction. The inflammation–oxidative stress cascade reaction represents the key pathological mechanism underlying radiation‐induced cardiac injury. Ionizing radiation‐induced endothelial cell damage activates multiple proinflammatory factors and inflammatory pathways, recruiting immune cells for inflammatory infiltration, leading to continuous amplification of inflammatory signals and subsequent ROS activation. Oxidative stress responses drive the activation of proinflammatory factors and key pathways through DNA damage and cellular senescence/death, synergistically promoting inflammation and collectively driving the vicious cycle of radiation‐induced cardiac injury.

The pathogenesis of RICD centers on a radiation‐triggered “inflammation–oxidative stress cascade” that establishes a self‐perpetuating cycle of cardiac damage (Figure 2). This cascade initiates when ionizing radiation (IR) directly injures the cardiac microvascular, damaging the phospholipid bilayer of vascular endothelial cell membranes and disrupting tight junction proteins (such as, ZO‐1/occludin). The resultant increase in vascular permeability and complement system activation creates a proinflammatory microenvironment [67]. Simultaneously, radiation‐damaged endothelial cells release damage‐associated molecular patterns (DAMPs), including high mobility group box 1 (HMGB1), S100 proteins, IL‐1α, and IL‐33. These endogenous danger signals activate innate immunity through pattern recognition receptors (Toll‐like receptors [TLRs], the receptor for advanced glycation end products, and NOD2 receptors) [68, 69], while concurrently stimulating multiple inflammatory pathways (NF‐κB, Smad, transforming growth factor‐β [TGF‐β], PI3K/Akt, GDF15, AMPK, and MAPK) [68, 70, 71]. A central process in RICD is the NOD‑like receptor protein 3 (NLRP3) inflammasome, which recognizes danger signals associated with pathogen infection or tissue damage [72, 73]. Upon activation, NLRP3 oligomerizes and recruits the adaptor protein ASC via PYD–PYD interactions, leading to Caspase‑1 activation and the subsequent release of the proinflammatory cytokines interleukin‑1β (IL‑1β) and IL‑18 [74, 75]. This initial inflammatory response is rapidly amplified through several parallel mechanisms: (1) upregulation of endothelial adhesion molecules (ICAM‑1/VCAM‑1), which promotes neutrophil and monocyte infiltration; (2) CCL2/CCR2‑mediated recruitment of M1‑polarized macrophages that secrete profibrotic factors (TGF‑β, PDGF, CTGF) [76]; and (3) activation of endoplasmic reticulum stress via the Sirt1/NF‑κB pathway, inducing the expression of stress markers (GRP78, CHOP, ATF6, Caspase‑12) [77, 78]. This multifaceted inflammatory cascade constitutes the initial phase of RICD pathogenesis and sets the stage for subsequent oxidative stress amplification and fibrotic remodeling.

Oxidative stress mechanisms exhibit distinct spatiotemporal dynamics. IR directly generates hydroxyl radicals (•OH), superoxide anion (O_2_•^−^), and hydrogen peroxide (H_2_O_2_), while mitochondrial electron transport chain uncoupling provides an additional source of superoxide. These reactive species are produced in large quantities and collectively assault myocardial tissue, leading to an accumulation of reactive oxygen species (ROS) that overwhelms physiological clearance mechanisms [79]. The resulting oxidative damage manifests as multiple molecular lesions: DNA single‑ and double‑strand breaks, base modifications, DNA–protein crosslinks, and dysregulation of lipid metabolism [80]. Concurrent suppression of the Nrf2‑mediated antioxidant defense system further exacerbates the oxidative–antioxidant imbalance [81]. This genomic instability triggers activation of the p53/p21 and p16 DNA damage response pathways and cell cycle arrest [82]. When repair mechanisms fail to resolve extensive damage, cells progress to either irreversible senescence or programmed cell death (PCD), releasing DAMPs and proinflammatory factors. Thus, oxidative damage begets inflammatory responses that further exacerbate tissue injury, establishing a vicious cycle that represents a fundamental mechanism of radiation‑induced cardiac pathology [83, 84].

The intricate interplay between inflammation and oxidative stress drives RICD pathogenesis through multiple interconnected mechanisms. At the molecular level, excessive ROS activate proinflammatory signaling via two principal routes: (1) NF‑κB activation through MAPK‑ and PI3K/Akt‑mediated phosphorylation of IKK and subsequent IκBα degradation, and (2) stimulation of the HMGB1/TLR4/MyD88 axis, which upregulates inflammatory mediators including cytokines, chemokines, and adhesion molecules [85]. Concomitantly, ROS‑damaged mitochondria promote the secretion of senescence‑associated secretory phenotype (SASP) factors, facilitating the formation of inflammatory senescent cells [86, 87]. These damaged mitochondria also activate the NLRP3 inflammasome, initiating the IL‑1β/IL‑6/C‑reactive protein (CRP) signaling cascade and enhancing hepatic CRP synthesis, thereby elevating cardiovascular risk [88]. The inflammatory milieu promotes extracellular matrix (ECM) degradation through the release of matrix metalloproteinases (MMPs) [89, 90], facilitating immune‑cell infiltration that further exacerbates oxidative stress through additional production of ROS and reactive nitrogen species (e.g., NO, superoxide, peroxynitrite) [91, 92]. Notably, TNF‑α exemplifies the bidirectional regulation: it enhances mitochondrial ROS by suppressing SOD2 and simultaneously activates NF‑κB/MAPK pathways to upregulate adhesion molecules (ICAM‑1/VCAM‑1) that promote leukocyte recruitment [93, 94]. Concurrently, proinflammatory cytokines impair antioxidant defenses by suppressing the activity of antioxidant enzymes (superoxide dismutase [SOD], catalase [CAT], glutathione peroxidase [GPx]) [95], while metabolic reprogramming toward glycolysis reduces the availability of NADPH for redox homeostasis [96]. These synergistic pathological processes culminate in cardiomyocyte apoptosis through both death‑receptor and mitochondrial pathways, triggering compensatory remodeling characterized by TGF‑β1‑mediated fibroblast activation via Smad2/3‑dependent and ‑independent mechanisms [97]. The resultant collagen deposition and myocardial fibrosis complete the self‑amplifying “inflammation–oxidative stress” circuit that ultimately leads to irreversible cardiac dysfunction. This comprehensive mechanistic understanding provides a rational basis for developing multitarget therapeutic strategies aimed at disrupting this vicious cycle.

### 3.3 Prevention and Treatments for RICD

In RICD, preventive strategies should be distinguished from therapeutic interventions for established cardiac injury. Prevention primarily aims to reduce cardiac radiation exposure and to suppress early oxidative and inflammatory injury before irreversible vascular remodeling, myocardial fibrosis, or functional decline develops, whereas treatment focuses on symptomatic or progressive cardiac dysfunction after injury has occurred. Clinically, radiation exposure is currently reduced mainly through procedural optimization—including lowering the frame rate from 25 to 3.75 fps, implementing ultra‑low dose fluoroscopy, and minimizing the detector‑to‑patient distance‐to alleviate radiation‑induced side effects [7, 8]. This strategy is simple, cost effective, widely applicable, and achieves substantial dose reduction, yet may compromise image quality and relies heavily on operator awareness and procedural discipline. The current clinical management to RICD remains predominantly focused on symptomatic treatment and complication management, lacking specific therapies that address the fundamental pathological processes driving disease progression [98]. This therapeutic gap has shifted research emphasis toward developing comprehensive, multitarget intervention strategies designed to simultaneously modulate multiple components of the inflammation–oxidative stress cascade that underlies RICD pathogenesis.

#### 3.3.1 Natural Drugs

Natural medicines possess unique therapeutic potential for the managements of RICD, largely owing to their multicomponent and multitarget characteristics (Table 1). Both traditional Chinese medicine (TCM) formulations and isolated bioactive plant compounds can synergistically modulate inflammatory and oxidative stress responses through coordinated actions on diverse signaling pathways. For instance, the classical herbal formula DangguiBuxue Tang (DBT) enhances myocardial antioxidant capacity via Nrf2 upregulation while suppressing NF‐κB‐mediated inflammatory signaling, a dual action that improves left ventricular systolic function and attenuates myocardial fibrosis [99]. Flavonoid compounds like zingerone demonstrate cardioprotection by reducing oxidative damage and inflammation, as well as by inhibiting Caspase‐3‐mediated apoptosis [100]. Similarly, curcumin protects cardiomyocytes through anti‐inflammatory and antifibrotic properties, while also enhancing the radiosensitivity of tumor cells [101, 102]. Furthermore, hesperidin provides vascular protection by suppressing inflammatory mediators release and oxidative stress‐induced endothelial dysfunction [103]. Other promising agents—including resveratrol‐rich black grape juice, L‐carnitine, and tanshinone—also mitigate radiation cardiotoxicity through combined antioxidant and anti‐inflammatory activities [104]. Despite these advantages—including favorable safety profiles and wide availability, challenges remain in fully realizing the clinical potential of natural medicines.

**TABLE 1:** Therapeutic strategies for RICD.

Category	Subtype	Drug name	Mechanism of action	References
Natural drugs	Chinese herbal medicine compound preparation	DangguiBuxue decoction (DBT)	Enhances left ventricular systolic function and reduces myocardial fibrosis	[99]
		Sheng‐Mai‐San (SMS)	Antioxidant effects	[105]
	Flavonoid compounds	Zingerone	Antioxidant and anti‐inflammatory	[106, 107]
		Turmerone	Attenuates oxidative stress, inflammation, and cardiac enzyme abnormalities	[100, 108]
		Curcumin	Suppresses oxidative stress, inflammation, and apoptotic DNA fragmentation	[109]
		Hesperidin	Inhibits inflammatory cytokine release and vascular permeability	[103]
	Grape polyphenols	Black grape juice (resveratrol, quercetin)	Provides antioxidant protection against radiation‐induced cardiac injury	[104, 110, 111, 112]
	Phenolic acid esters	CAPE (caffeic acid phenethyl ester)	Antioxidant and anti‐inflammatory	[113, 114]
	Amino acid derivatives	L‐carnitine	Promotes β‐oxidation of fatty acids, alleviates myocardial oxidative stress	[110]
	Diterpenoid quinones	Tanshinone	Inhibits myocardial injury via p38/p53 axis	[115]
	Pineal hormone	Melatonin	Provides antiapoptotic, antioxidant, anti‐inflammatory	[116]
Chemical compounds	ACE inhibitors	Captopril	Inhibits ACE, improves capillary function, reduces myocardial fibrosis	[102, 117]
	Angiotensin II receptor Blocker	Losartan	Blocks AT_1_ receptors and inhibits TGF‐β/Smad fibrosis pathways	[70]
	Statins	Atorvastatin	Suppresses TGF‐β/Smad3/CTGF and NF‐κB inflammatory signaling	[118]
	Organic thiophosphate radioprotectors	Amifostine	Scavenges radiation‐induced ROS and protects cellular organelles	[119, 120]
	Alkaloids	Colchicine	Upregulates IL‐10, while inhibiting TGF‐β expression	[121, 122]
	PPAR‐α agonists	Fibrates	Activates PPAR‐α and suppresses NF‐κB‐mediated inflammation	[123]
	Combination therapy	Pentoxifylline + α‐tocopherol	Downregulates TGF‐β; improves LV function; fibrosis rebound may occur after withdrawal	[124, 125]
	Targeted small molecules	Sestrin2 activator IPW‐537	Inhibit oxidative stress and fibroblast activation; reduce fibrosis	[126, 127, 128]
		miRNA‐21 inhibitor	Inhibit oxidative stress and fibroblast activation; reduce fibrosis	[126, 127, 128]
	Immunomodulatory agents	Thalidomide	Blocks TNF‐α, IL‐6, and TGF‐β signaling pathways	[129]
Antibodies and peptides	Recombinant growth factors	rhNRG‐1β	Activates ErbB2/4 receptors, triggers PI3K/AKT and ERK signaling	[130]
		GDF‐11	Restore metabolic homeostasis and attenuate oxidative stress through AMPKα pathway activation	[131]
	Peptide hormone	Adropin	Activates VEGFR2/PI3K/Akt pathway, downregulates NOX4, eNOS, Caspase‐3 expression	[87, 132]
	Cytokine antagonist	Anakinra	Blocks IL‐1R, inhibits CCL2/CCL5	[133]
	IL‐6 antibody	Ziltivekimab	Specifically blocks IL‐6 signaling, avoids neutropenia	[134]
	Recombinant antioxidant Enzyme	SOD/catalase	Directly scavenges ROS, protects cardiomyocytes	[135, 136]
Nanomedicine delivery systems	Liquid metal core–shell	LMN	X‐ray‐triggered diselenide bond cleavage; polymer shell releases drugs; stabilizes mitochondria	[137]
	Natural compounds with nanocarriers	Ganoderma lucidum spore oil (GSLO)	Improves solubility and stability; reduces radiation‐induced ROS; protects mitochondria	[138]
	Plant‐derived delivery platforms	Se@CMCM	Regulates MnSOD and MDA levels, modulates selenoprotein expression, and promotes adaptive immune responses via T cell and M2 macrophage polarization	[80]
	Conventional drug with nanocarriers	Metformin	Activates AMPK; inhibits CXCL1‐mediated fibroblast recruitment	[139]
Hydrogels	Natural	Alginate, chitosan, dECM, collagen, gelatin, HA, fibrin, matrigel	Biocompatible; low cytotoxicity; simulate ECM microenvironment	[140, 141, 142]
	Synthetic	PEG, PAA derivatives, PVA	Tunable mechanical properties; low immunogenicity; batch consistency	[143, 144, 145, 146]
	Hybrid	PEG–HA‐SH conductive hydrogel	Simulates ECM mechanics; carries ADSCs and DNA‐eNOS; promotes tissue repair	[147, 148]

#### 3.3.2 Compound‐Based Drugs

Pharmacological interventions for RICD primarily involve cardiovascular drugs and targeted antioxidants that precisely modulate key components of the inflammation–oxidative stress cascade (Table 1). Renin–angiotensin–aldosterone system inhibitors form the cornerstone of RICD management. Captopril, for example, ameliorates postradiotherapy microvascular dysfunction, reduces fibrosis, and delays disease progression [102, 117]. Losartan affords biphasic protection by inhibiting both classical and nonclassical fibrotic pathways [70]. Statins confer additional benefits through pleiotropic, nonlipid effects, such as, suppressing NF‐κB and inhibiting inflammatory adhesion molecule [118]. Radioprotectors represent another vital strategy for preventing radiation injury and are a major research focus, akin to the therapeutic approaches described above. Amifostine, an approved radioprotective agent, enhances coronary perfusion and preserves cardiac output by scavenging free radicals, protecting mitochondrial, and maintaining endothelial integrity [118]. Furthermore, combined therapy with pentoxifylline and α‐tocopherol shows promise in preserving left ventricular function during radiotherapy, partly through the downregulation of TGF‐β [124, 125]. Synthetic compounds offer distinct advantages, including well‑characterized structures, clear mechanisms of action, and scalable production capacity. Consequently, the development of next‑generation compounds with improved selectivity and the optimization of therapeutic regimens have become focal points in clinical research.

#### 3.3.3 Antibody and Peptide‐Based Therapeutics

Antibody and peptide‐based therapeutics have gained significant attention as innovative treatment options for RICD (**Table** **1**), owing to their exceptional targeting specificity, precise molecular recognition, favorable biocompatibility, and potent regulatory functions. Recombinant Neuregulin‐1β (rhNRG‐1β) attenuates cardiomyocyte apoptosis and dysfunction through activation of the PI3K/AKT and ERK signaling pathways [74]. When administered during radiotherapy, rhNRG‐1β significantly reduces both the incidence and severity of radiation‐associated cardiac damage [149]. Similarly, growth differentiation factor 11 has demonstrated therapeutic potential by restoring metabolic homeostasis and attenuating oxidative stress through AMPKα pathway activation [131]. The peptide hormone adropin exerts antifibrotic effects by modulating the VEGFR2/PI3K/Akt signaling pathway to suppress the expression of NOX4, eNOS, and Caspase‐3 [132]. The cardiac protease corin has been identified as a critical regulator of radiation‐induced cardiomyocyte senescence that promotes RICD progression, whilst natriuretic peptide overexpression downregulates corin and improves cardiac outcomes [87]. Despite these promising developments, the clinical translation of antibody and peptide therapeutics faces considerable challenges, including manufacturing complexity, formulation stability, and restricted administration options.

#### 3.3.4 Nanomedicine Drug Delivery Systems

Nanotechnology has revolutionized therapeutic approaches for RICD through the development of advanced drug delivery systems (**Table** **1**). These nanoscale platforms offer superior cardiac targeting, enhanced tissue compatibility, and reduced systemic toxicity while protecting therapeutic payloads from premature degradation and clearance‐significantly improving drug bioavailability and retention time at target sites. A groundbreaking example is the liquid metal‐based core–shell nanomedicine, featuring a gallium metal core and multifunctional polymer shell with unique radiopaque properties, X‐ray shielding capability, and radiation–responsive antioxidant activity. This innovative design enables controlled drug release by X‐ray‐induced cleavage of diselenide bonds in the polymer matrix, effectively mitigating radiation‐induced myocardial damage, preserving mitochondrial function, and preventing fibrotic remodeling [137]. Besides, integration of natural compounds with nanocarriers has emerged as a particularly promising strategy. Ganoderma lucidum spore oil (GLSO) has potent antioxidant properties but suffers from poor solubility and stability. Chen et al. addressed these limitations by developing GLSO@P188/PEG400 NS, which demonstrate enhanced water solubility and biocompatibility while effectively scavenging radiation‐induced ROS and protecting mitochondrial integrity [138]. Plant‐derived delivery platforms represent another innovative approach, combining sustainability with therapeutic efficacy. The Se@CMCM platform, developed using Cordyceps militaris as a bioactive carrier for selenium delivery, demonstrates remarkable cardioprotection against radiation damage through multiple mechanisms: maintaining redox homeostasis via regulation of manganese SOD (MnSOD) and MDA levels, modulating selenoprotein expression, and promoting beneficial immune responses through T cell and M2 macrophage polarization [80].

Injectable hydrogel represent a cutting‐edge approach in tissue engineering, offering dual functionality as both biomechanical scaffolds and delivery vehicles for therapeutic agents. These minimally invasive systems uniquely replicate the ECM while providing mechanical support to damaged myocardium and enabling localized release of bioactive factors and cells to enhance tissue regeneration [150]. Current hydrogels platforms fall into three categories: natural, synthetic, and hybrid hydrogels. Natural polymer hydrogels (e.g., alginate, chitosan, dECM, collagen) demonstrate excellent biocompatibility and ECM‐mimicking properties, though their clinical translation is limited by mechanical weakness and potential immunogenicity [151]. Synthetic alternatives address these limitations through tunable physical properties (mechanical strength, porosity, gelation kinetics) and superior batch‐to‐batch consistency [140]. Hybrid hydrogels synergize these advantages by combining natural polymers’ bioactivity with synthetic materials’ engineering precision, creating optimized platforms with cell‐adhesive motifs, appropriate mechanical properties, and tailored degradation profiles for cardiac applications [143]. Notable innovations include conductive hybrid hydrogels incorporating ADSCs and eNOS‐loaded nanocomposites, which enhance both degradation behavior and cell/drug adhesion compared with conventional PEG systems [147]. Further functionalization through nanoparticle integration (polymeric, metallic, ceramic) enables multifunctional hydrogel systems with expanded therapeutic capabilities [140]. Despite their promise in cardiac repair, hydrogel applications specifically for RICD remain under explored, warranting focused investigation to establish their efficacy in radiation‐damaged myocardium.

Collectively, each therapeutic strategy for RICD management presents distinct advantages and limitations. Natural medicines offer the benefits of multitarget synergy and favorable safety profiles; however, their clinical translation is hindered by compositional complexity, standardization difficulties, and incomplete mechanistic understanding. Synthetic pharmaceuticals have established efficacy in practice but are constrained by limited target specificity and safety concerns. Antibody and peptide therapies enable precise molecular targeting, yet their clinical application faces obstacles, such as, manufacturing complexity, formulation instability, and restricted dosing options. Nanomedicine delivery systems provide a transformative platform that enhances cardiac targeting, reduces systemic toxicity, and improves drug bioavailability; nevertheless, their long‑term biosafety and scalable manufacturing require systematic evaluation. The combination of nanodelivery systems with antibodies or peptides may represent the future trajectory of precision therapeutics. Currently, RICD has become one of the significant factors limiting the clinical application of radiotherapy. A thorough understanding of its core pathological mechanism—the “inflammation–oxidative stress cascade”—is essential for developing effective intervention strategies. Traditional treatment approaches have demonstrated limited efficacy, whereas natural pharmaceutical preparations, synthetic compounds, protein‑ and peptide‑based drugs, and nanomedicine delivery systems each offer unique characteristics, providing diverse options for the prevention and treatment of RICD. With advances in multidisciplinary integration, breakthroughs will be achieved in the diagnosis, prevention, and treatment of RICD, offering stronger assurance for the long‑term quality of life of radiotherapy patients.

## 4 Radiation‐Induced Lung Disease

Alveoli are the fundamental structural units that sustain pulmonary gas exchange [152]. Radiation triggers multiple modes of death in alveolar epithelial cells, disrupts the balance between autophagy and cellular repair, and compromises alveolar architecture, ultimately driving the transition from acute radiation pneumonitis to late pulmonary fibrosis. Consequently, single‑target therapies directed against individual cell‑death pathways are often insufficient to halt disease progression. Multitarget combination strategies and precision drug‑delivery systems better reflect the intertwined nature of these cell‑death processes and may also reduce treatment‑related adverse effects.

### 4.1 Epidemiology of RILI

RILI is a serious complication of radiotherapy, characterized by progressive respiratory dysfunction that may ultimately lead to cor pulmonale and contribute significantly to noncancer mortality in treated patients [152, 153]. This condition manifests as a biphasic pathological process, beginning with acute radiation‐induced pneumonitis (RIP) and progressing to chronic radiation‐induced lung fibrosis (RILF). The reported incidence varies substantially (5–58%) across clinical studies [154, 155], reflecting differences in diagnostic criteria and follow‐up durations. Current clinical management faces significant challenges owing to several factors: the complex pathophysiology of RILI, nonspecific early symptoms, and delayed radiographic changes that typically 2–8 weeks after symptom onset [156, 157]. These limitations create a narrow diagnostic window and underscore the critical need for better understanding of molecular mechanisms to develop targeted therapies.

The progression from acute RIP to RILF involves a cascade of events initiated by IR‐induced activation of PCD pathways in as spatiotemporally regulated manner [158, 159]. During the acute phase (<3 months postradiation), extensive apoptosis of Type I pneumocytes (which constitute 90–95% of alveolar epithelium) [160] triggers compensatory proliferation of Type II pneumocytes. However, this reparative process becomes dysregulated during the sub‐acute phase (3–6 months), with abnormal Type II cell activation leading to surfactant production defects and further epithelial damage [161]. Concurrently, IR causes direct injury to the pulmonary microvascular endothelium, resulting in three major pathological effects: (1) suppression of fibrinolytic activity, (2) increased vascular permeability, and (3) excessive release of proinflammatory mediators [162, 163]. These changes collectively impair microcirculatory function, cause tissue hypoxia, and disrupt the critical endothelial–epithelial barrier—all hallmark features of RIP [164]. PCD triggers inflammatory cascade escalation through DAMP release, including HMGB1 and adenosine triphosphate, which activate the TLRs/NLRP3 inflammasome signaling axis [165, 166]. This activation recruits and stimulates neutrophils, macrophages, and lymphocytes, creating a self‐perpetuating cycle of injury through multiple mechanisms: (1) promoting myofibroblast differentiation and ECM remodeling and (2) establishing a ROS–HIF‐1α‐mediated positive feedback loop that sustains inflammation and drives fibrotic progression during the chronic phase (>6 months) [167, 168]. This intricate pathophysiology explains both the clinical progression of RILI and the current limitations in its management.

### 4.2 Pathogenesis of RILI

**FIGURE 3:**
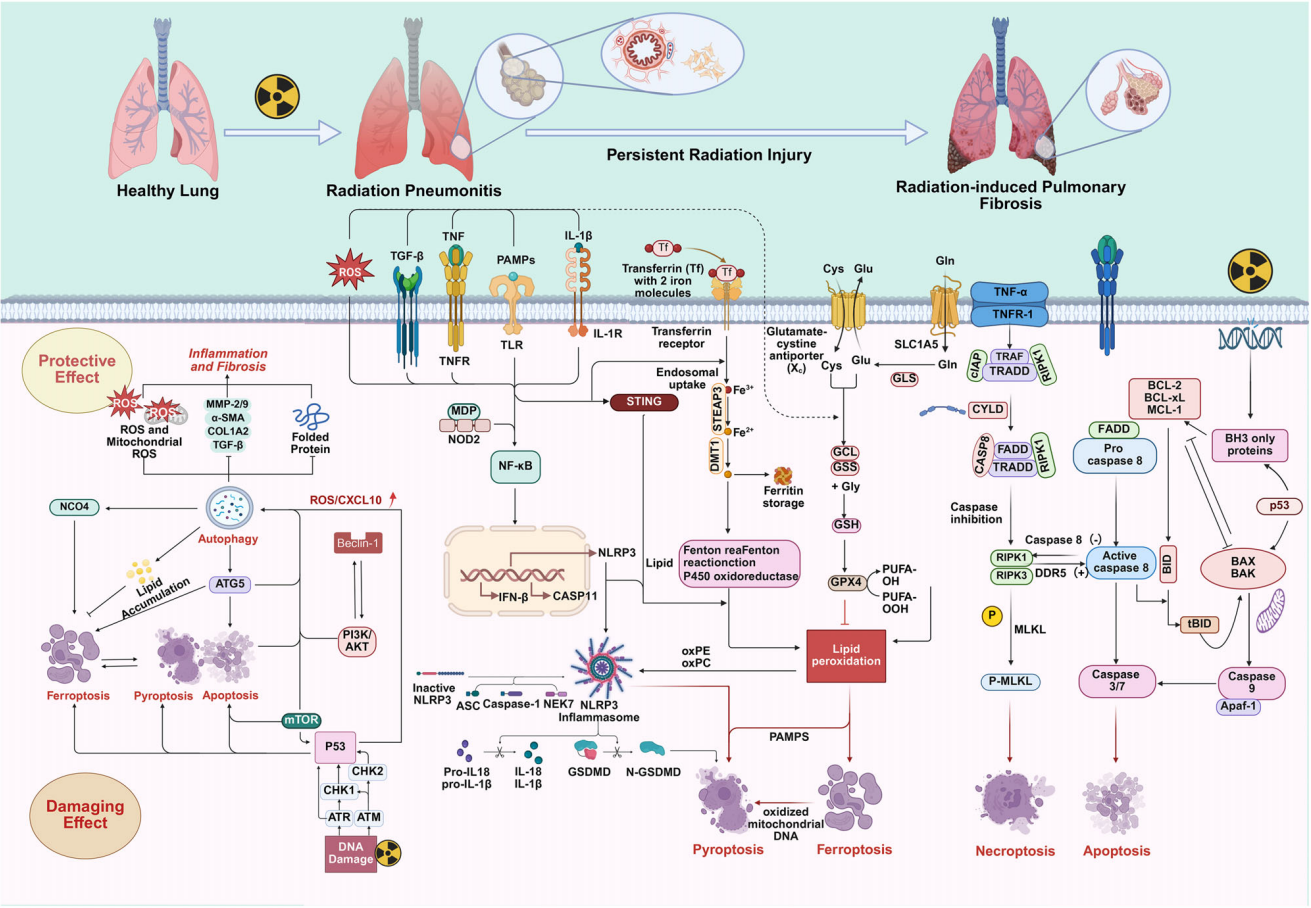
Complex dynamic interactive networks of programmed cell death pathways in radiation‐induced lung injury. Multiple programmed cell death pathways constitute a complex signaling network that promotes the development and progression of radiation‐induced pulmonary injury. Inhibition of either apoptosis or necroptosis activates alternative death pathways. Ferroptosis and pyroptosis jointly participate in lipid peroxidation processes, synergistically exerting profibrotic effects. Autophagy serves as a double‐edged sword, capable of clearing damaged mitochondria and proteins to maintain microenvironmental homeostasis while simultaneously promoting abnormal protein degradation that accelerates cell death. Abbreviations: PAMPs: pathogen‐associated molecular patterns; TNFR: tumor necrosis factor receptor; CHK1: checkpoint kinase 1; ATR: ataxia telangiectasia and Rad3‐related protein; MDP: muramyl dipeptide; IFN‐β: interferon beta; CASP11: Caspase‐11; NEK7: NIMA‐related kinase 7; N‐GSDMD: N‐terminal gasdermin D; Tf: transferrin; STEAP3: six‐transmembrane epithelial antigen of prostate 3; DMT1: divalent metal transporter 1; SLC1A5: solute carrier family 1 member 5; GLS: glutaminase; GCL: glutamate–cysteine ligase; GSS: glutathione synthetase; PUFA‐OH: hydroxy polyunsaturated fatty acid; PUFA‐OOH: hydroperoxy polyunsaturated fatty acid; oxPE: oxidized phosphatidylethanolamine; oxPC: oxidized phosphatidylcholine; TNFR1: tumor necrosis factor receptor 1; TRAF: TNF receptor‐associated factor; TRADD: TNFR1‐associated death domain protein; cIAP: cellular inhibitor of apoptosis protein; CYLD: CYLD lysine 63 deubiquitinase; FADD: Fas‐associated death domain protein; CASP8: Caspase‐8; p‐MLKL: phosphorylated mixed lineage kinase domain‐like protein; MCL‐1: myeloid cell leukemia 1; BH3: BCL‐2 homology 3; BID: BH3‐interacting domain death agonist; tBID: truncated BID; APAF‐1: apoptotic protease‐activating factor 1.

The pathophysiological process of RILI involves a sophisticated network of interacting PCD pathways that demonstrate dynamic cross‐regulation within the irradiated tissue microenvironment [169, 170]. Rather than functioning independently, these PCD mechanisms engage in complex crosstalk, with the potential for interconversion depending on cellular context and molecular signaling dynamics (Figure 3).

#### 4.2.1 Molecular Interconversion Between Apoptosis and Necroptosis

IR induces cellular damage through both direct effects on macromolecules (DNA, proteins) and indirect effects via radiolytic byproducts, ultimately triggering apoptotic cell death [171]. The apoptotic cascade is initiated through Caspase‐8 or ‐9 activation, which subsequently activates executioner Caspases‐3/7 [172]. The tumor suppressor p53 serves as a central regulator of radiation‐induced apoptosis in lung epithelium, coordinating multiple proapoptotic pathways including PARP, BAX, HLO‐1, and TGFβ/SMAD, while simultaneously promoting cellular senescence via p21 activation [173]. In contrast, necroptosis represents a caspase‐independent form of PCD mediated through receptor‐interacting protein kinase 3 (RIPK3)‐mediated phosphorylation of the mixed lineage kinase domain‐like protein (MLKL) [174]. In RILI, necroptosis activation correlates strongly with Tnfrsf10b gene upregulation [175]. These two death modalities differ fundamentally in their inflammatory consequences: apoptosis typically proceeds without significant inflammation, while necroptosis induces robust inflammatory responses through cellular membrane rupture and release of intracellular contents [172, 174].

Experimental evidence reveals a finely balanced relationship between apoptosis and necroptosis in RILI pathogenesis. Radiation‐induced oxidative stress and mitochondrial damage concurrently activate both pathways during the acute injury phase [176]. Inhibition studies demonstrate remarkable plasticity between these death‐mechanisms‐Caspase‐8 suppression leads to increased RIPK1/3 activation and MLKL phosphorylation, effectively converting apoptotic signals to necroptosis [177, 178]. Conversely, RIPK3 inhibition can redirect cell fate toward apoptosis [179, 180]. This bidirectional conversion capacity has led to the proposed “threshold model” of apoptosis–necroptosis regulation, where cellular energy status, redox balance, and key protein expression levels collectively determine the dominant death pathway [176]. These findings provide crucial insights for developing more effective therapeutic strategies that account for the dynamic interplay between PCD mechanisms in RILI.

#### 4.2.2 Synergistic Effect of Ferroptosis and Pyroptosis

Emerging research has established ferroptosis and pyroptosis as critically interconnected PCD pathways in RILI [176]. Ferroptosis, an iron‐dependent nonapoptotic death mechanism, is regulated through three core metabolic pathways: (1) GSH metabolism, (2) lipid metabolism, and (3) iron homeostasis. The antioxidant enzyme GPX4 serves as the central regulator of radiation‐induced ferroptosis, with its functional decline leading to catastrophic lipid peroxidation and subsequent cell death [181]. Notably, these accumulated lipid peroxides serve dual pathological roles—they represent hallmark biomarkers of ferroptosis [132], while simultaneously activating the NLRP3 inflammasome and promoting GSDMD cleavage to initiate pyroptosis [182]. Pyroptosis, characterized by inflammatory caspase activation (both Caspase‐1‐dependent and ‐independent variants), mediates robust inflammatory responses through GSDMD‐mediated pore formation and subsequent release of IL‐1β and IL‐18 [171]. Lipidomic analyses have identified radiation‐induced oxidation of specific phospholipids (cardiolipin and phosphatidylserine) as key molecular bridges between these pathways [183]. These oxidized lipid species not only indicate ferroptotic stress but also directly trigger pyroptosis by inducing FL‐GSDME oligomerization and membrane targeting [183, 184]. The synergistic amplification of lung injury occurs through multiple mechanisms: (1) Shared upstream activators (STING pathway, TGF‐β1 signaling, ROS) simultaneously induce both death pathways [185, 186, 187]. (2) Ferroptotic release of oxidized mitochondrial DNA (mtDNA) acts as DAMP to propagate pyroptosis in neighboring cells. (3) The resulting “death spreading effect” creates a self‐reinforcing cycle of cellular injury and inflammation [186]. The synergistic proinjury effects of ferroptosis and pyroptosis offer new insights into the interactive mechanisms between various forms of PCD in RILI.

#### 4.2.3 Dual Effects of Autophagy

Autophagy, serves as a critical cellular stress response mechanism that exhibits context‐dependent effects in RILI, functioning as both a protective pathway and a potential contributor to tissue damage [170, 188]. Under physiological conditions, radiation‐induced autophagy plays a beneficial role by eliminating damaged mitochondria and misfolded proteins that generate ROS, while simultaneously modulating inflammatory responses to mitigate radiation damage [189]. This protective function is evidenced by autophagy's ability to downregulate key fibrotic mediators including α‐smooth muscle actin (α‐SMA), COL1A2, TGF‐β1, and MMPs (MMP‐2/9), along with its capacity to maintain mitochondrial homeostasis through selective mitophagy, thereby reducing mitochondrial ROS production and preventing DNA damage accumulation [189, 190]. However, the persistent oxidative stress characteristic of RILI can transform autophagy from a protective mechanism into a pathological process. Excessive autophagic activity, triggered by radiation‐induced CXCL10 overexpression and ROS accumulation in natural killer cells, leads to the lysosomal degradation of NKG2D receptors. This process significantly impairs natural killer cell function and exacerbates the progression of radiation pneumonitis [191]. The complex regulatory network of autophagy involves key molecular players, such as, mTOR, p53, and ATG5, which serve as critical nodes connecting different PCD pathways. These proteins function as molecular switches that can modulate apoptosis, pyroptosis, and other cell death mechanisms depending on cellular context and microenvironmental conditions [192, 193].

The interaction between autophagy and ferroptosis demonstrates particular complexity in RILI pathogenesis. On one hand, nuclear receptor coactivator 4‐mediated ferritinophagy promotes lysosomal degradation of ferritin, increasing intracellular free iron levels and consequently enhancing cellular susceptibility to ferroptosis [194]. On the other hand, autophagy can paradoxically confer resistance to ferroptosis by facilitating intracellular lipid accumulation [195]. These seemingly contradictory findings highlight the dynamic and phase‐dependent nature of autophagy regulation in RILI, explaining the variable outcomes observed in clinical trials of autophagy modulators. The temporal evolution of RILI from acute inflammation to chronic fibrosis likely requires distinct autophagy modulation strategies, suggesting that therapeutic approaches must be carefully tailored to the specific disease phase. This nuanced understanding of autophagy's dual roles provides a crucial foundation for developing targeted interventions in RILI management.

### 4.3 Prevention and Treatments for RILI

For RILI, by far the most widely deployed protection remains lead‑based full‑ or partial‑body medical shielding, which directly safeguards both operators and patients. In recent years, diverse lead‑free alternatives have emerged, including polymer composites, bismuth, tungsten, rare‑earth materials, aerogels, electrospun membranes, coatings, molded materials, and 3D‑printed structures, aiming to overcome the drawbacks of lead, such as, toxicity, high density, rigidity, and poor breathability [2, 3]. As another vital strategy for preventing radiation injury, radioprotectors represent a major research focus‐similar as the therapeutic strategies.

#### 4.3.1 Single‐Target Interventions

The growing recognition of PCD network complexity in RILI has catalyzed a fundamental transformation in treatment strategies, shifting from isolated single‐target interventions to integrated multitarget approaches. Current pharmacological research has yielded three major classes of targeted therapies: (1) apoptosis‐modulating agents that preserve various cell populations through diverse signaling pathways [110, 196, 197], (2) ferroptosis inhibitors acting primarily via Nrf2 pathway suppression and GPX4 upregulation [198, 199, 200, 201], and (3) pyroptosis‐targeting compounds like ACT001, VX‐765, and MCC950 that employ multiple mechanisms to inhibit NLRP3 inflammasome activity [202, 203]. While these targeted therapies show preclinical promise, they frequently encounter clinical limitations due to the interconnected nature of PCD pathways. A compelling example is Nec‐1 (RIPK1 inhibitor), which successfully suppresses necroptosis by reducing MLKL phosphorylation but unexpectedly enhances apoptotic cell death—a phenomenon termed “death pathway switching” that exemplifies the compensatory mechanisms undermining single‐pathway interventions [204]. Similarly, autophagy modulators demonstrate context‐dependent efficacy, with hydrogen‐rich solutions showing benefit through AMPK/mTOR/ULK1‐mediated autophagy activation [205], while NVP‐AUY922 achieves protection via autophagy inhibition and GPX4 elevation. These findings collectively underscore the inherent limitations of unilateral PCD modulation and highlight the necessity for network‐level therapeutic strategies that can simultaneously regulate multiple nodal points within the interconnected PCD pathways [206].

#### 4.3.2 Novel Perspectives on Combined Intervention Strategies

The recognized limitations of single‐target approaches have propelled the development of innovative combination strategies for managing RILI, founded on two fundamental therapeutic principles: multitarget synergy and precision delivery systems (Table 2). Sequential intervention strategies leverage our understanding of the dynamic progression of RILI, employing multitarget regimens that address the evolving dominance of specific cell death pathways. This approach is exemplified by Deng et al.’s work with engineered flagellin (FlaA N/C), which capitalizes on the molecular interplay between inflammasome activation and autophagy to suppress pyroptosis while promoting protective inflammatory autophagy [193]. Similarly, Wu et al. demonstrated the efficacy of melatonin in concurrently modulating apoptosis and autophagy pathways, resulting in improved mitochondrial function and reduced oxidative stress in radon‐exposed lung tissue [207]. The power of simultaneous multipathway intervention is further evidenced by combination therapies targeting necroptosis and apoptosis, where dual‐drug regimens have shown superior efficacy to monotherapies in whole‐body irradiation models [208]. Particularly striking results were achieved with a three‐drug combination (JP4‐039, Necrostatin‐1, and baicalein), which markedly improved survival rates while normalizing inflammatory cytokine profiles [209]. However, these promising outcomes must be balanced against the need for careful optimization of dosing regimens and thorough evaluation of potential synergistic toxicities. Complementing these multitarget strategies, precision delivery systems represent a breakthrough in precision medicine for RILI. Advanced nanoplatforms, such as, virus‐like particles engineered to codeliver ferroptosis and apoptosis inhibitors, have demonstrated remarkable tissue specificity and therapeutic efficacy. The SOD@ARA290‐HBc system exemplifies this approach, achieving localized protection of alveolar epithelium through combined antiapoptotic and antiferroptotic actions while modulating macrophage polarization [210].

**TABLE 2:** Therapeutic agents for RILI.

Category	Subtype	Drug name	Mechanism of action	References
Single‐target therapy	Necroptosis inhibitors	Nec‐1	Inhibit MLKL phosphorylation	[211]
	Ferroptosis inhibitors	Ferrostatin‐1	Inhibit GPX4 degradation	[198]
		Dihydroartemisinin	Inhibit the Nrf2/HO‐1 pathway	[199]
		Vitamin C	Inhibit the MAPK signaling pathway	[196]
		Liproxstatin‐1	Eliminate ROS, activate the Nrf2 pathwayrestore GPX4 levels	[200]
		Ferrocene‐appended GPX4 inhibitors	Inhibit GPX4 activity	[201]
		Crocetin	Downregulation of Tnfrsf10b	[212]
	Autophagy inhibitors	APS	Inhibit Caspase‐3 and Bax	[213]
		NVP‐AUY922	Inhibit GPX4‐related autophagy	[206]
		Azithromycin	Inhibit the production of proinflammatory cytokines	[214]
	Autophagy enhancer	Ulinastatin	Enhancement of TGF‐β1 and LC3, and reduction of COL1A1 and COL1A2	[190]
	Apoptosis	Lovastatin	Inhibit Rho pathway	[215]
		Silibinin	Inhibition of Caspase‐9 and Caspase‐3 activity	[216]
		AEOL 10150	Reduction of monocyte/macrophage infiltration	[217]
		LET‐bFGF	Protect vascular endothelial cells	[218]
		Anisodamine (654‐2)	Activate the Nrf2/ARE pathway	[219]
		MPLA	Promote the polarization of RAW 264.7 cells toward the M1 phenotype	[25]
		GSPs	Inhibit TGF‐b1/Smad3/Snail	[220]
		CLA	Inhibit macrophage infiltration, alveolar fibrosis	[221]
		GSTP1	Inhibit MAPK, NF‐κB activation	[222]
		AST	Inhibit Bax and Caspase‐3	[116]
		Astilbin	Reduction of p53 acetylation	[223]
		GTS‐21	Inhibit HMGB1/TLR4/NF‐κB	[224]
		LC	Reduction in the secretion of IL‐1β, IL‐6, and TNF‐α	[225]
		THP	Reduction of BALF cell recruitment and decrease in BALF protein levels	[226]
		PQQ	MOTS‐c‐dependent mechanisms protect mitochondria	[227]
	Pyroptosis	ACT001	Inhibit NLRP3	[215]
		Pulmozyme	cGAS/STING/NLRP3	[202]
		Andrographolide	AIM2 inflammasome	[203]
		VX‐765	Inhibit Caspase‐1	[198]
		MCC950	Inhibit NLRP3 Inflammasome	[213]
Combination therapy	Autophagy, apoptosis	Melatonin	Improves mitochondrial function and reduces oxidative stress	[207]
	Autophagy, pytoptosis	FlaA N/C	Capitalizes on the molecular interplay	[193]
	Necroptosis, apoptosis	JP4‐039, Necrostatin‐1	Antinecroptosis after antiapoptosis 48 h	[208]
		JP4‐039, Necrostatin‐1, and baicalein	Normalizes inflammatory cytokine profiles	[209]
	Necroptosis, pyroptosis	MSC‐Lipo‐OPC	Inhibit neutrophil	[228]
		Forsythiaside A+UVI3003	Inhibit TLR4/MAPK/NF‐κB, MLCK/MLC2	[229]
	Apoptosis, ferroptosis	SOD@ARA290‐HBc	Reduce ROS production and improve mitochondrial function	[210]
	Ferroptosis, pyroptosis	VX‐765 + Ferrostatin‐1	Inhibit GPX4, Caspase‐1	[198]

Current therapeutic strategies for RILI fall into two main categories: single‐target interventions and combination therapies, each with distinct outlooks. While single‐target approaches offer specificity for particular PCD pathways, their efficacy is limited by compensatory activation within interconnected PCD networks [209]. In contrast, combination strategies show greater clinical potential by multitarget synergy and precision delivery systems. Examples include sequential multitarget regimens [209] and triple‐drug combinations that have significantly enhance survival rates [209], overcoming key limitations of single‐pathway interventions. However, these advanced strategies still face significant challenges, such as, optimization of dosing schedules, assessment of synergistic toxicity, and off‐target effects. Precision delivery systems, including nanoplatforms that enable tissue‐specific targeting for localized protection, are promising but remain constrained by suboptimal delivery efficiency and a lack of long‐term safety data [209]. Together, these emerging paradigms highlight the need for system‐level approaches that address the complex interplay of PCD pathways in RILI. Advancing the field will require solving the challenges notes above to realize truly precision interventions. The insights garnered from such efforts may not only improve outcomes in RILI but also inform the treatment of other diseases driven by dysregulated cell death, heralding a new era in the management of complex pathological processes.

## 5 Radiation‐Induced Skin Injury

The skin, the body's outermost barrier, is one of the tissues most frequently exposed during radiotherapy. Radiation‐induced skin injury (RISI) is clinically characterized by persistent, nonhealing lesions and a progressive transition from acute inflammation to chronic fibrosis. Fibroblast activation is central to the maintenance of chronic inflammation and fibrosis; it continuously promotes immune cell recruitment and polarization, thereby contributing to impaired wound healing. In turn, the dysregulated immune microenvironment sustains fibroblast activation and pathological matrix remodeling, further driving cutaneous fibrosis. Accordingly, therapeutic strategies may target TGF‑β signaling and senescent cells to terminate aberrant fibrotic programs, while mesenchymal stem cells (MSCs) and their secreted products can modulate the local immune microenvironment and promote tissue repair. Because the skin is readily accessible for local intervention, emerging biomaterials—including hydrogels, nanomaterials, and three‐dimensional bioprinting platforms—have been widely explored for the treatment of RISI.

### 5.1 Epidemiology of RISI

RISI represents a frequent complication observed in both nuclear accidents and therapeutic radiation, with approximately 95% of patients undergoing radiotherapy experiencing varying degrees of cutaneous damage [230]. The National Cancer Institute's Common Terminology Criteria for Adverse Events classifies RISI into distinct acute and chronic phases based on temporal progression and pathological characteristics [231].

Acute‐phase manifestations typically develop during radiation treatment and persist for up to 90 days postexposure, characterized primarily by robust inflammatory responses. This phase features prominent immune cell infiltration (lymphocytes, macrophages, and neutrophils) into irradiated skin tissues, accompanied by substantial release of proinflammatory cytokines and chemokines that exacerbate local inflammation. Clinically, this inflammatory cascade manifests as erythema, edema, pain, and fever, with severe cases progressing to vesiculation, alopecia, and desquamation that significantly compromise skin barrier function. Persistent inflammation serves as the driving force behind the transition to chronic RISI, a process that may continue evolving over months to years [230]. The chronic phase pathophysiology centers on fibroblast‐to‐myofibroblast transdifferentiation, marked by excessive production of α‐SMA and collagen deposition [232]. This fibrotic transformation induces extensive ECM remodeling [233], ultimately leading to characteristic chronic manifestations including skin thickening, atrophy, pigmentation abnormalities, telangiectasia, adnexal structure reduction, and ulcer formation [232]. Beyond physical symptoms, such as, pain, pruritus, and ulceration, RISI carries significant psychological consequences, frequently resulting in social withdrawal, anxiety, and depression that collectively impair patients' quality of life [230]. Current clinical management predominantly relies on antibiotics, corticosteroids, and nonsteroidal anti‐inflammatory drugs, though establishing definitive treatment efficacy remains challenging due to the absence of standardized quantitative assessment methods and insufficient large‐scale clinical trials [234].

A more comprehensive understanding of RISI pathogenesis, particularly the molecular mechanisms underlying chronic fibrotic progression, is essential for developing novel therapeutic strategies. Such advances could significantly improve both clinical outcomes and quality of life for affected patients while addressing the current limitations in evidence‐based treatment approaches.

### 5.2 Pathogenesis of RISI

#### 5.2.1 Fibroblast Activation and Senescence Axis

IR exposure induces remarkable fibroblast heterogeneity through the coordinated activation of multiple cellular sources, including skin‐resident, BM‐recruited subpopulations, and newly generated mesenchymal‐like cell populations via epithelial–mesenchymal transition (EMT) and endothelial–mesenchymal transition (EndMT) [232, 233]. TGF‐β emerges as the master regulator of this heterogeneous fibroblast population, orchestrating both proliferation and pathological activation through multiple downstream signaling cascades. Under radiation conditions, TGF‐β exerts pleiotropic effects through three major mechanisms: (1) enhancing protein synthesis and fibroblast proliferation via PI3K/AKT/mTOR axis activation, (2) serving as a potent chemotactic factor for fibroblast recruitment to injury sites [235, 236], and (3) promoting EMT to expand the myofibroblast pool [237]. The fibrotic potential of TGF‐β exhibits dose‐dependent characteristics, with its signaling initiated through TβRII receptor binding and subsequent Smad2/3–Smad4 complex formation that directly activates profibrotic gene expression (α‐SMA, fibronectin, collagen) [238, 239]. TGF‐β further amplifies its effects through synergistic crosstalk with Wnt/β‐catenin signaling, where induced Wnt ligands promote β‐catenin nuclear translocation to enhance myofibroblast differentiation while simultaneously stabilizing Smad proteins to create a positive feedback loop [240, 241]. Additional complexity arises from TGF‐β’s activation of MAPK pathways (ERK, JNK, p38), which regulate fibrosis‐related gene transcription through both Smad‐dependent and independent mechanisms, thereby intensifying inflammatory responses and perpetuating fibroblast activation within the fibrotic niche [232]. The fibrotic cascade is reinforced through downstream mediators, particularly connective tissue growth factor (CTGF/CCN2), which amplifies TGF‐β’s effects via dual Smad‐dependent and independent pathways to drive fibroblast proliferation and ECM production [242, 243]. MMPs (MMP‐1, MMP‐3) and AP‐1 transcriptional complex activation further contribute to ECM remodeling, while cooperative interactions between TGF‐β/Smad and AP‐1 complexes enhance expression of key fibrotic components (c‐JUN, IL‐11, fibronectin, Collagen Iα2) [244, 245].

**FIGURE 4:**
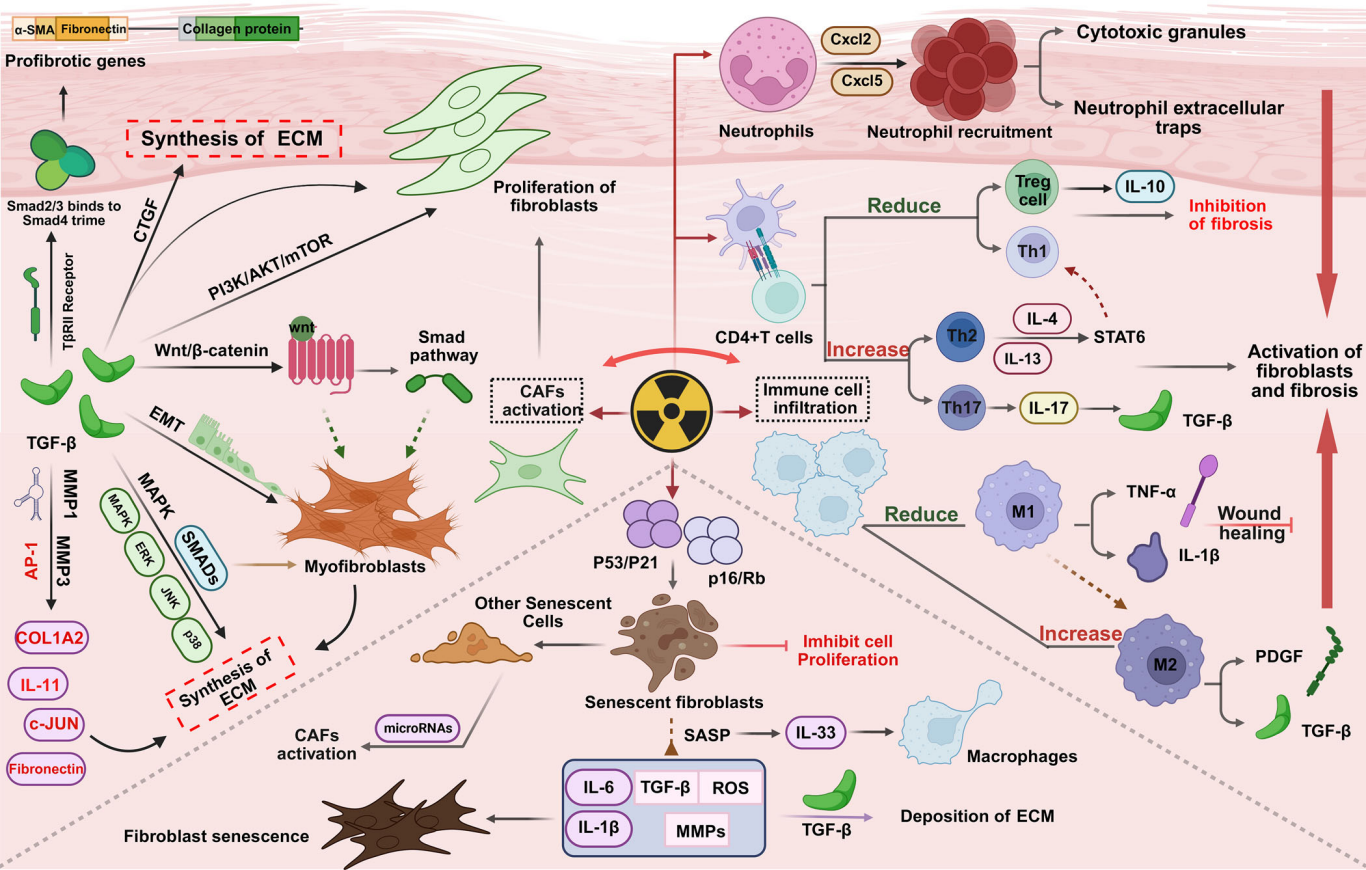
Dual‐axis driving mechanism of chronic fibrosis. Fibroblasts and immune cells synergistically participate in the pathological progression of radiation‐induced skin injury. Sustained activation, proliferation, and senescent death of fibroblasts constitute the core factors driving fibrotic progression in radiation‐induced skin injury. Multiple immune cell infiltration and phenotypic imbalances also represent key processes participating in RISI. These two mechanisms mutually influence each other, collectively promoting the progression of radiation‐induced skin injury.

Radiation‐induced cellular senescence, mediated through p53/p21 and p16/Rb pathways, represents a paradoxical response that both limits proliferation and promotes fibrosis via the SASP [246, 247]. This complex secretome includes proinflammatory cytokines (IL‐6, IL‐1β), fibrogenic factors (TGF‐β), matrix‐remodeling enzymes (MMPs), and ROS that collectively influence wound healing and fibrosis progression [248]. While SASP components like IL‐33 can modulate macrophage function to aid tissue repair [247], they simultaneously drive myofibroblast differentiation and ECM deposition through TGF‐β signaling [248]. The senescent phenotype spreads through bystander effects, exacerbating tissue damage via paracrine senescence induction [249], while exosomal microRNAs (e.g., miR‐21) from senescent cells reinforce fibrotic signaling by suppressing Smad7 expression and enhancing TGF‐β pathway activity [250]. Together, these mechanisms establish a self‐perpetuating fibrotic microenvironment where senescent and activated fibroblasts play central roles in radiation‐induced skin fibrosis pathogenesis (Figure 4).

#### 5.2.2 Immune Cell Infiltration and Dynamic Imbalance Axis

Emerging evidence reveals a critical dichotomy in macrophage function during RISI, where resident macrophages maintain tissue homeostasis while infiltrating macrophages drive fibrotic progression [232]. The initial radiation exposure triggers M1 macrophage polarization, characterized by proinflammatory cytokine production (TNF‐α, IL‐1β) that initiates wound healing responses. However, as inflammation persists, a pathological shift toward M2 dominance occurs, marked by excessive secretion of fibrogenic factors (TGF‐β, PDGF) that stimulate fibroblast activation and ECM deposition [251, 252]. Therefore, the balance between M1 and M2 macrophages is crucial for maintaining tissue homeostasis. In the context of RISI, the extensive release of signaling molecules (such as, IL‐4, IL‐13, and IL‐10) disrupts this balance, leading to a predominance of the M2 phenotype. This macrophage polarization imbalance toward M2 transformation not only promotes ECM deposition but also inhibits effective tissue remodeling and repair, creating a sustained fibrotic environment. This polarization imbalance, driven by elevated IL‐4, IL‐13, and IL‐10 signaling, creates a self‐perpetuating fibrotic microenvironment that simultaneously promotes ECM accumulation and impairs tissue regeneration [253, 254].

The immune dysregulation extends to T cell populations, where radiation induces a profound Th2/Th17 bias accompanied by Th1 and Treg suppression [255]. Th2‐derived IL‐4 and IL‐13 activate STAT6 signaling to stimulate collagen production while counteracting Th1‐mediated antifibrotic effects. Concurrently, Th17 cells exacerbate fibrosis through IL‐17‐mediated inflammation and TGF‐β induction [256]. The compromised function of Treg cells, normally protective through IL‐10 secretion, allows unfettered progression of fibrotic responses [257, 258]. Neutrophils further amplify tissue damage through Cxcl2/Cxcl5‐mediated recruitment and subsequent release of cytotoxic granules and neutrophil extracellular traps [259], highlighting the multifaceted immune contribution to fibrosis [260].

**FIGURE 5:**
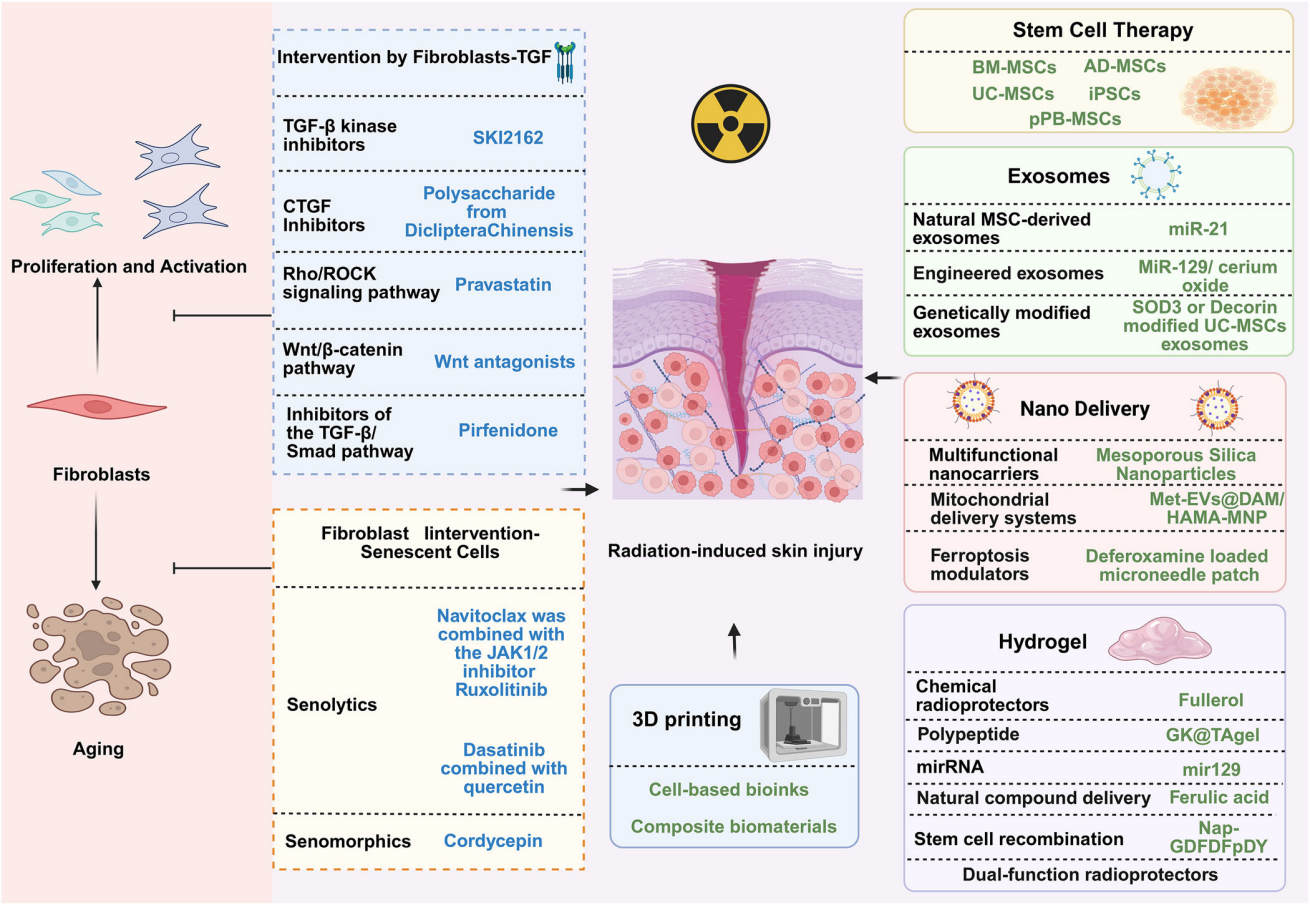
Therapeutic strategies for radiation‐induced skin injury. Current research on fibroblast intervention strategies primarily focuses on the TGF‐β pathway and senescent cells. And the development of novel materials and emerging technologies—including stem cells, exosomes, nanodelivery systems, hydrogels, and 3D printing technology—has provided innovative therapeutic approaches for the treatment of radiation‐induced cutaneous injury. Abbreviations: iPSCs: induced pluripotent stem cells; JAK1/2: Janus kinases 1 and 2; Met‐EVs: metformin‐induced, mitochondria‐enriched extracellular vesicles; DAM: decellularized adipose matrix; HAMA: hyaluronic acid methacrylate; MNP: microneedle patch.

The core pathophysiology of chronic radiation‐induced fibrosis centers on reciprocal interactions between the fibroblast and immune axes. Activated fibroblasts secrete chemotactic factors that perpetuate immune cell infiltration and polarization, while conversely, the altered immune milieu drives persistent fibroblast activation and pathological ECM remodeling (Figure 5). This bidirectional crosstalk creates a vicious cycle that underlies the therapeutic resistance observed in RISI. Elucidating the molecular mediators of this fibroblast–immune network will be crucial for developing targeted interventions capable of disrupting the fibrotic cascade while preserving tissue regenerative capacity.

### 5.3 Prevention and Treatments for RISI

For RISI, prevention occupies a particularly important position because cutaneous injury is frequent, visible, and often begins during the radiotherapy course. Preventive strategies include reduction of unnecessary skin dose, physical or topical barrier protection [2, 3] before persistent fibroblast activation and chronic fibrosis develop.

#### 5.3.1 Fibroblast Intervention Strategies

The central role of TGF‐β in RISI progression has established it as a primary therapeutic target, with multiple intervention strategies showing promise in modulating fibroblast function. Small molecule kinase inhibitors (SKI2162, vactosertib, galunisertib, LY2109761) attenuate fibrosis through specific inhibition of TGF‐β1 kinase activity, while the neutralizing peptide P144 reduces radiation‐induced muscular fibrosis through competitive binding to the TGF‐β1 Type I receptor [261]. Beyond direct TGF‐β inhibition, therapeutic strategies now encompass downstream pathway modulation. For example, the polysaccharide from Dicliptera chinensis exerts protective effects through CTGF pathway inhibition [243], while statins achieve similar results via Rho/ROCK signaling [262]. Clinically available agents like pirfenidone mediate repair through TGF‐β/Smad pathway blockade [263], and soluble Wnt receptors (sLRP6E1E2) or antagonists alleviate fibrosis by targeting the Wnt/β‐catenin cascade [264]. Additional opportunities exist through, antioxidant and anti‐inflammatory approaches that regulate fibroblast activity and immune microenvironment homeostasis. Emerging therapeutic approaches are increasingly focused on cellular senescence, advancing two distinct strategies. The first involves senolytics—such as, navitoclax, ruxolitinib, and dasatinib, quercetin combinations—which selectively clear senescent cells and reduce SASP‐mediated damage [262]. The second employs senomorphics, like cordycepin, which modulate senescence‐associated pathways (e.g., Nrf2, AMPK) without cell elimination [262]. Both strategies show therapeutic promise for mitigating radiation‐induced cutaneous injury, particularly through their targeting of myofibroblast differentiation and senescence.

#### 5.3.2 Stem Cell Therapeutic Approaches

MSCs have emerged as a promising therapeutic approach for RISI due to their unique multipotent differentiation capacity, immunomodulatory properties, and paracrine effects [230].Currently, the three primary MSC sources being investigated include BM‐derived (BM‐MSCs), adipose tissue‐derived (AD‐MSCs), and umbilical cord‐derived (UC‐MSCs) stem cells, with therapeutic applications focusing on three main modalities: direct cell infusion, exosome‐based therapy, and genetically modified approaches. Among these, AD‐MSCs have gained particular clinical traction due to their accessibility and favorable safety profile [265]. BM‐MSCs have been shown to ameliorate radiation‐induced skin fibrosis through TGF‐β receptor‐dependent modulation of local inflammation [266, 267]. UC‐MSCs exhibit greater proliferative capacity than BM‐MSCs and promote tissue repair by activating the AKT‐mediated upregulation of HIF‐1α/β.This mechanism underpins their anti‐inflammatory, antioxidant, proangiogenic, and autophagy‐enhancing effects [268, 269]. The recognition that MSC therapeutic effects are primarily paracrine mediated [185] has spurred development of cell‐free alternatives. MSC‐derived exosomes (MSC‐Exo) carry bioactive miRNAs that regulate HIF‐1 and PTEN/PI3K/AKT pathways, facilitating epithelial repair, immune modulation, and reduced vascular damage [270, 271]. Engineered exosomes further improve targeting through the incorporation of drugs, growth factors, or gene‐editing tools [272]. Genetic engineering approaches have expanded the therapeutic potential of MSCs through several key strategies: (1) enhanced efficacy via overexpression of therapeutic genes (SOD3, decorin, HGF) [273, 274]; (2) improved homing capacity through HMGB1 modification that increases CXCR4 expression and endothelial differentiation [275]; (3) targeted delivery using pPB peptide‐functionalized MSCs that specifically bind PDGFRβ while maintaining native MSC functions [276]; and (4) prolonged survival through HGF overexpression to enhance persistence in injury microenvironments [277]. Together, these advances—from native MSC infusions to engineered exosomes and genetically enhanced cells—mark significant progress in the treatment of RISI.

#### 5.3.3 Applications of Biomaterials

The continuous advancement of biomaterials has significantly expanded therapeutic options for RISI. Among these, hydrogels have emerged as particularly promising candidates due to their exceptional water retention, biocompatibility, and ECM‐mimicking 3D porous structure [272]. Modern hydrogel systems are now engineered with diverse functional components—such as, fullerenol, sildenafil citrate, GK@TAgel peptides, VEGF, miRNA‐129, TCM monomers, MSCs (Nap‐GDFDFpDY), and nanomaterials like molybdenum disulfide [272]—to overcome mechanical limitations of conventional hydrogels and deliver multifunctional therapeutic benefits, including antimicrobial, antioxidant, anti‐inflammatory, angiogenic, and tissue regenerative properties. Innovative hydrogel designs now incorporate dual radioprotective mechanisms [278]. For example, the nano‐GDY@SH system—comprising nanographdiyne particles within sodium hyaluronate hydrogel—provides both physical shielding via high water content and chemical protection through potent ROS scavenging [279]. Nanotechnology has further revolutionized RISI treatment by enhancing drug delivery. Advanced systems based on mesoporous silica, cerium oxide, and chitosan nanoparticles significantly improve targeting and bioavailability, while microneedle platforms enable controlled mitochondrial delivery to wound sites [280]. A major step forward comes from smart responsive biomaterials that react to environmental cues—such as, temperature, pH [281], photothermal stimuli, or biochemical markers (H_2_O_2_, enzymes, glucose)—allowing spatiotemporally precise drug release [282]. Photothermal‐responsive materials are especially notable for their deep tissue penetration using NIR light, as demonstrated by gold nanorod–gallic acid bioadhesives that provide controlled antioxidant release [283]. Additionally, 3D bioprinting has opened new frontiers in tissue engineering for RISI. Customizable bioinks enable patient‐specific solutions that synergistically combine therapeutic effects. Applications range from radiation damage assessment using 3D‐printed fibroblast constructs [284] to autologous cell‐based wound repair materials that accelerate healing [285].

Owing to these novel materials and emerging technologies, treatment options for radiation‐induced cutaneous injury are more diverse than those for other organ systems (Figure 5). Small molecule inhibitors show definitive antifibrotic effects in preclinical models [261]; however, because their targets (e.g., TGF‐β and Wnt) are extensively involved in immune‐regulation and tissue homeostasis, systemic inhibition risks unpredictable adverse effects. Clinical translation is further challenged by issues of drug stability, tissue compatibility, and potential toxicities. While antisenescence agents introduce novel therapeutic concepts [286], their optimal dosing and therapeutic windows require refinement. Cell‐based therapies similarly confront translational obstacles, including potential tumorigenic risk and cellular preparation heterogeneity that influences dose consistency and therapeutic outcomes [230]. These issues underscore the imperative for further investigation to optimize MSC sourcing, culture conditions, quality standards, and treatment protocols. Exosomes, offer a cell‐free alternative with advantages in tissue penetration and targeted delivery [270, 271, 287, 288], yet production standardization and scalable manufacturing remain significant bottlenecks. Biomaterial platforms—especially hydrogel systems—enable multimodal therapy by integrating pharmaceuticals, nanomaterials, and stem cells (SCs) [272], showing particular promise for RISI. Nevertheless, substantial challenges persist in vascularization, cell sourcing, material biocompatibility, and optimizing printing technology to ensure the long‐term viability and clinical efficacy of bioprinted tissues. Overall, the most promising clinical strategy appears to be an integrative approach combining genetically engineered MSCs or exosomes with stimuli‐responsive hydrogels. This leverages biomaterials to overcome delivery barriers while harnessing the multifaceted reparative mechanisms of cells or vesicles for synergistic effects. Moving forward, all emerging therapies will require rigorous quality standards, comprehensive pharmacokinetic and long‐term safety data, standardized potency assays, and predictive biomarkers of efficacy to successful transition from laboratory to the clinic.

## 6 Radiation‐Induced Hematological Toxicity

The hematopoietic system is composed primarily of hematopoietic stem and progenitor cells, which are characterized by rapid proliferation and sustained self‑renewal. Their reliance on accurate DNA replication and repair, together with intact mitochondrial function, underpins long‑term hematopoiesis and contributes substantially to the pronounced radiosensitivity of this system [36, 289]. Radiation‑induced DNA damage and mitochondrial dysfunction reinforce one another, further promoting oxidative stress, inflammatory activation, cellular senescence, and cell death, and ultimately driving hematopoietic stem‑cell exhaustion and persistent hematopoietic dysfunction. Accordingly, current therapeutic strategies focus on protecting and repairing DNA, preserving mitochondrial integrity, and restoring or reconstructing the hematopoietic system. Combination therapies that intervene at multiple stages—from initial molecular damage to overt hematopoietic failure—may therefore hold considerable potential for clinical development.

### 6.1 Epidemiology of Radiation‐Induced Hematological Toxicity

The hematopoietic system's heightened sensitivity to radiation stems from its proliferative nature and self‐renewal capacity, making it particularly vulnerable to radiation‐induced damage [36]. Radiation‐induced hematological toxicity (RIHT) manifests in two distinct forms: (1) single high‐dose exposures induced H‐ARS that rapidly damage BM microenvironments, characterized by precipitous declines in platelets, granulocytes, and lymphocytes [36]; (2) repeated low‐dose radiation induced chronic hematologic injury that leads to cumulative BM damage [289]. The severity of RIHT follows a dose‐dependent pattern, with whole‐body exposures >1 Gy typically inducing H‐ARS, while accumulated doses of 2–3.5 Gy cause persistent BM suppression [290]. Notably, moderate‐to‐high radiation doses inflict long‐term hematopoietic impairment through sustained reductions in SC reserves and self‐renewal capacity [291]. Globally, approximately 15% of radiotherapy patients experience varying degrees of hematopoietic dysfunction [292], underscoring the critical need to elucidate RIHT pathogenesis [292]. IR primarily targets nuclear DNA, creating dose‐dependent damage patterns [293, 294], while simultaneously compromising mtDNA integrity and disrupting electron transport chain assembly [295]. When DNA repair capacity is overwhelmed, ATM kinase activation triggers the p53–PUMA pathway, inducing proapoptotic Bcl proteins (BAX, NOXA, PUMA) to mediate mitochondrial outer membrane permeabilization and initiate hematopoietic SC (HSC) apoptosis [296, 297, 298]. Radiation‐induced mitochondrial dysfunction generates persistent ROS that exacerbates nuclear DNA damage [299], while released mtDNA activates the cGAS–STING inflammatory pathway via VDAC1 oligomerization [79, 300, 301], creating a destructive feedback loop.

This dual nuclear‐mitochondrial damage cascade drives progressive hematopoietic collapse through multiple mechanisms. Lymphocytes, exhibiting extreme radiosensitivity, undergo rapid p53/BCL‐2‐mediated apoptosis, while megakaryocyte differentiation arrest from GATA1 epigenetic silencing causes delayed thrombocytopenia (∼60% reduction by Day 14) [292]. As compensatory mechanisms fail, residual HSCs accelerate differentiation, depleting long‐term repopulating pools (“SC exhaustion paradox”) while impaired mitophagy permits toxic metabolite accumulation [302]. Surviving HSCs undergo metabolic reprogramming and clonal expansion influenced by mitochondrial genome heterogeneity [301], upregulating MHCII to recruit BM Tregs that paradoxically promote neighboring HSC senescence through PKA activation [303]. The resulting SASP disrupts MSC differentiation balance, while LOXL2‐mediated fibrosis and adipocyte expansion create an increasingly hostile microenvironment that spatially constrains and functionally exhausts remaining HSCs [304, 305, 306, 307]. Localized radiotherapy further exacerbates this damage by inducing IL‐1/IL‐18‐mediated macrophage and CD8^+^ T cell proliferation that perpetuates HSC depletion [308]. Ultimately, this multifaceted assault on genomic stability and microenvironmental homeostasis establishes an intractable cycle of progressive hematopoietic failure [309].

### 6.2 Pathogenesis of RIHT

#### 6.2.1 DNA Damage as the Initial Event of RIHT

**FIGURE 6:**
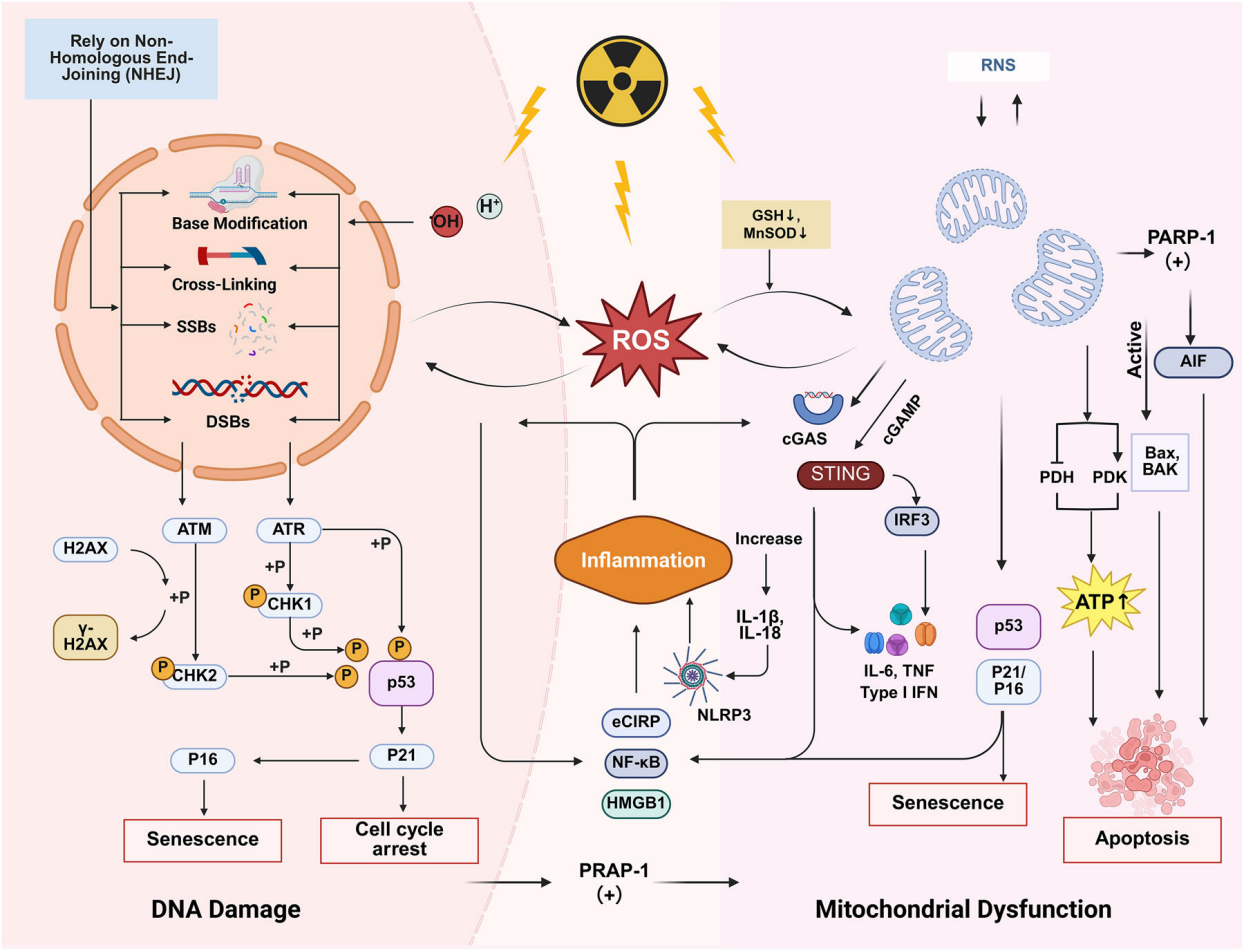
Molecular mechanisms of radiation‐induced hematological toxicity: the interactive network of DNA damage and mitochondrial dysfunction. The interplay between exogenous radiation damage and endogenous DNA lesions creates a molecular foundation for persistent hemato‐toxicity. The mitochondrial disturbances create a vicious cycle of hematopoietic toxicity through mtDNA‐mediated inflammatory activation, persistent oxidative stress, programmed cell death induction, and accelerated senescence. The intricate interplay between DNA damage and mitochondrial dysfunction lies at the heart of the mechanism driving the amplification of radiation‐induced hematological toxicity. Abbreviation: IRF3: interferon regulatory factor 3.

The development of radiation‐induced hematotoxicity involves a complex cascade process originating from DNA damage and exacerbated by mitochondrial dysfunction (Figure 6). At the molecular level, radiation induces DNA damage through two distinct but interrelated mechanisms. The direct effect occurs when radiation energy is absorbed by DNA molecules, causing structural alterations, including base modification, cross‐linking, single‐strand breaks, and double‐strand breaks [310]. Among these lesions, DSBs are particularly detrimental due to their potential for erroneous repair leading to genomic information loss [311], making them the primary mediator of radiation‐induced hematopoietic cell death [312]. A characteristic feature of IR is the production of complex DNA DSBs, also known as “clustered DNA damage,” which refers to DSBs accompanied by additional oxidative base damage, abasic sites, SSBs, or other DSBs within a 10‐base pair region [310, 313]. These complex lesions are repaired with significantly reduced efficiency compared with isolated breaks, resulting in persistent genomic instability [314].

The cellular response to DNA damage is orchestrated by the ATM kinase, which serves as the primary sensor for DSBs [124]. Following radiation exposure, ATM undergoes phosphorylation and activation, leading to the formation of γ‐H2AX foci at damage sites—a well‐established biomarker for DSBs [315]. The indirect effect of radiation involves radiolysis of water molecules, generating ROS, particularly hydroxyl radicals (·OH), that subsequently attack DNA [316, 317]. ROS‐induced DNA damage triggers ATM‐mediated phosphorylation of p53, upregulating p21 expression and inducing cell cycle arrest, senescence, or apoptosis [318]. The nature of DNA damage varies significantly with radiation quality: high‐LET radiation (neutrons, α‐particles) tends to cause more complex direct DNA damage, while low‐LET radiation (X‐rays, γ‐rays) primarily induces damage through radical‐mediated indirect effects [319].

The cellular response to DNA damage is further complicated by the inherent limitations of DNA repair systems. Two major pathways exist for DSB repair: the error‐prone nonhomologous end joining (NHEJ) mechanism that operates throughout the cell cycle, and the more accurate but slower homologous recombination pathway restricted to S/G2 phases [320, 321]. The quiescent nature of HSCs, which predominantly reside in G0 phase, predisposes them to rely on NHEJ for DNA repair, increasing their susceptibility to genomic instability [321]. This repair pathway bias, combined with the accumulation of repair errors, progressively elevates the mutational burden and may initiate malignant transformation [289], creating a self‐perpetuating cycle of genomic instability. An often‐overlooked aspect of radiation‐induced hematotoxicity is the significant contribution of endogenous DNA damage, which includes hydrolytic damage (∼10,000 base losses/cell/day), deamination, oxidative lesions (tens of thousands daily), and alkylation damage [322]. This background damage synergizes with radiation‐induced lesions to overwhelm cellular repair capacity, leading to repair system saturation and accelerated hematopoietic dysfunction. The interplay between exogenous radiation damage and endogenous DNA lesions creates a molecular foundation for persistent hematotoxicity, characterized by cumulative genomic instability and progressive functional decline in the hematopoietic system.

#### 6.2.2 Mitochondrial Dysfunction Increases RIHT

Beyond nuclear DNA damage, mitochondrial impairment plays a pivotal role in the pathogenesis of RIHT through multiple interconnected mechanisms. Radiation exposure triggers immediate mitochondrial membrane permeability changes, initiating a cascade of events mediated by voltage‐dependent anion channel (VDAC) 1 oligomerization. This process forms outer membrane pores that facilitate mtDNA release into the cytoplasm [323], where it activates the cGAS–STING pathway. The subsequent production of cyclic GMP–AMP (cGAMP) stimulates Type I interferon (IFN‐I) responses, contributing to BM damage and hematopoietic suppression. As the primary cellular source of ROS, dysfunctional mitochondria significantly amplify radiation injury through sustained oxidative stress. Radiation generates excessive ROS and reactive nitrogen species via mitochondrial mechanisms [324, 325], creating a prolonged oxidative burden that persists for months postexposure. This oxidative insult is exacerbated by the concurrent impairment of critical antioxidant defenses, including MnSOD and GSH systems [326, 327], creating a dangerous imbalance in redox homeostasis. The metabolic consequences of radiation‐induced mitochondrial dysfunction are particularly profound in HSCs, which rely on precise mitochondrial regulation for proper function. Radiation induces significant metabolic reprogramming characterized by suppressed pyruvate dehydrogenase (PDH) activity and upregulated PDH kinase (PDK) expression [328], effectively starving mitochondria of acetyl‐CoA for ATP production. Metabolomic analyses reveal dramatic perturbations in lipid metabolism, with elevated short‐chain acylcarnitines and ketone bodies contrasting with depleted long‐chain acylcarnitines [329, 330]. These metabolic disruptions critically impair the balance between damage repair and SC maintenance, severely compromising hematopoietic recovery. Radiation further exacerbates hematopoietic dysfunction through mitochondrial‐mediated activation of apoptotic and senescent pathways. The intrinsic apoptosis pathway is triggered by Bax/Bak‐mediated outer membrane permeabilization, cytochrome *c* release, and caspase cascade activation. PARP‐1 hyperactivation depletes cellular NAD^+^ and ATP pools, facilitating apoptosis‐inducing factor (AIF) nuclear translocation and caspase‐independent cell death. Radiation further exacerbates hematopoietic dysfunction through mitochondrial‐mediated activation of apoptotic and senescent pathways. The intrinsic apoptosis pathway is triggered by Bax/Bak‐mediated outer membrane permeabilization, cytochrome *c* release, and caspase cascade activation. PARP‐1 hyperactivation depletes cellular NAD^+^ and ATP pools, facilitating AIF nuclear translocation and caspase‐independent cell death [331]. Simultaneously, radiation‐induced respiratory complex II dysfunction generates excessive superoxide that activates the ROS/p53/p21/p16 axis, driving cellular senescence [332]. NO contributes significantly to this process by promoting DNA damage and inflammation, with its effects modulated by arginine availability and inducible NO synthase activity [333]. Collectively, these mitochondrial disturbances create a vicious cycle of hematopoietic toxicity through: (1) mtDNA‐mediated inflammatory activation; (2) persistent oxidative stress; (3) metabolic dysfunction; (4) PCD induction; (5) accelerated senescence. This multifaceted mitochondrial involvement underscores its central role in both the acute and chronic manifestations of radiation‐induced hematotoxicity, presenting multiple potential targets for therapeutic intervention. The sustained nature of these mitochondrial alterations helps explain the prolonged hematopoietic suppression observed following radiation exposure and highlights the need for strategies that address both nuclear and mitochondrial aspects of radiation damage.

#### 6.2.3 The Vicious Cycle of Damage Amplification Through DNA Damage and Mitochondrial Dysfunction Interaction Networks

The intricate interplay between DNA damage and mitochondrial dysfunction lies at the heart of the mechanism driving the amplification of RIHT toxicity. IR induces DNA double‐strand breaks, which in turn stimulate mitochondrial biogenesis via the ATM–AMPK–PGC1α signaling pathway [304, 334]. However, this compensatory response often fails to counteract the extensive damage inflicted by radiation. The hyperactivation of PARP1, triggered by DNA damage, results in severe NAD^+^ depletion, aggravating the mitochondrial energy crisis [335]. Concurrently, excessive ROS generated by impaired mitochondrial respiratory chain function diffuse into the nucleus, where oxidative products, such as, 8‐hydroxyguanosine, exacerbate nuclear DNA damage. The disrupted crosstalk between mitochondria and the nucleus further complicates this process, hindering the cell's ability to balance resource allocation between DNA repair and mitochondrial maintenance [305].

This damage amplification is further perpetuated by a vicious cycle of inflammatory signaling and oxidative stress. The NF‐κB signaling network plays a pivotal role in this mechanism, being modulated both by ATM phosphorylation (initiated by DNA damage) and mitochondria‐derived ROS [336, 337]. Upon activation, NF‐κB regulates a multitude of target genes involved in DNA repair, cell cycle checkpoints, mitochondrial antioxidants, and cytokine production, thereby reinforcing an inflammatory feedback loop. The systemic impact of this response is mediated through circulating DAMPs, including extracellular cold‐inducible RNA‐binding protein and HMGB1 [338], which activate the innate immune system and provoke widespread immune reactions. Additionally, cytoplasmic mtDNA binds to and activates the NLRP3 inflammasome, triggering the excessive release of proinflammatory cytokines, such as, IL‐1β and IL‐18. These factors not only induce pyroptosis but also exacerbate damage to the hematopoietic microenvironment [339].

Under sustained oxidative stress and chronic inflammation, a significant proportion of HSCs undergo senescence and apoptosis, further amplifying the detrimental effects. The self‐perpetuating cycle formed by DNA damage and mitochondrial dysfunction not only elucidates the complexity and persistence of RIHT but also offers critical insights for developing targeted therapeutic strategies. Interventions designed to disrupt key nodes within the DNA–mitochondrial damage network—particularly those targeting critical signaling pathways—hold substantial clinical promise for mitigating postradiation hematological injury.

### 6.3 Prevention and Treatments for RIHT

For RIHT, prevention focuses on protecting the BM and HSC niche before cumulative DNA and mitochondrial damage becomes irreversible. Therefore, dose‐sparing strategies and radioprotectors that scavenge ROS, enhance DNA repair, preserve mitochondrial function, or support hematopoietic regeneration should be considered preventive when used before or during irradiation.

#### 6.3.1 Protective and Repair Strategies for DNA and Mitochondrial Damage

Pharmacological strategies for mitigating radiation‐induced hematotoxicity primarily focus on protecting and repairing DNA damage through free radical scavenging and enhanced repair mechanisms [340]. Melatonin, as a natural antioxidant, markedly reduces radiation‐induced γ‐H2AX foci formation and inhibits apoptosis whilst preserving hematopoietic function through upregulation of G‐CSF expression and modulation of the p53–PUMA pathway [294, 341]. Similarly, other natural antioxidants—including grape seed proanthocyanidin extract [342], resveratrol [343], and various polysaccharide compounds [344]—exert protective effects against radiation‐induced hematotoxicity by suppressing cellular apoptosis. Synthetic compounds, such as, 5‐methoxytryptamine–α‐lipoic acid, derived from melatonin and α‐lipoic acid, also demonstrate hematopoietic protection postirradiation through antioxidative mechanisms [345]. Additionally, 5‐androstenediol, a key radioprotective agent for the hematopoietic system, not only exhibits antioxidant activity but also mitigates radiation‐induced apoptosis and enhances DNA repair processes [346]. Plant‐derived compounds, such as, cantharidin and GPx, confer radioprotection by enhancing antioxidant enzyme activity, including SOD and CAT, thereby reducing DNA damage in BM cells [347]. Recent advances in drug development have highlighted DNA repair enhancers as a promising therapeutic avenue. For instance, BIO 300, a ginkgo biloba glycoside preparation, activates key DNA repair proteins, such as, p53, ATM, CHK2, and BRCA1, making it a potential candidate for treating acute radiation syndrome and delayed radiation effects [348]. Epigenetic modulators, such as, the histone deacetylase inhibitor phenylbutyric acid and the polyphenolic acetate compound 7,8‐diacetoxy‐4‐methylthiocoumarin, offer novel approaches for radiation injury repair through regulation of histone acetylation, DNA damage repair, and modulation of hematopoietic stem and progenitor cell maintenance and self‐renewal [349].

Mitochondrial protection and functional maintenance represent another critical therapeutic approach, encompassing mitochondrial antioxidants, mtDNA–cGAS–STING pathway modulation, and mitochondrial protectants. Mitochondrial‐targeted antioxidants, such as, the nitroxide compound JP4‐039, function as SOD mimetics, reducing oxidative stress and BM cell death while improving survival rates following lethal‐dose irradiation [350]. Nanomaterial‐based strategies, including mitochondrial‐targeted cerium oxide nanoclusters (TPP‐PCNL), selectively localize to mitochondria and mitigate nuclear DNA damage caused by mitochondrial oxidative stress, thereby preserving genomic integrity [351]. The mechanistic investigations reveal that VDAC inhibitor DIDS has been shown to elevate BMNC and HSC levels in irradiated mice, suggesting its potential for preventing or treating H‐ARS [323]. Natural compounds like theaflavins promote nuclear factor erythroid 2‐related factor 2 (NRF2) nuclear translocation, upregulating antioxidant enzymes, such as, heme oxygenase‐1, NAD(P)H quinone oxidoreductase 1, and SOD2, thereby reducing oxidative stress in HSCs [352]. Collectively, these therapeutic strategies targeting DNA repair and mitochondrial dysfunction provide a robust theoretical and practical framework for the clinical management of radiation‐induced hematotoxicity.

#### 6.3.2 Recovery and Reconstruction Strategies for the Hematopoietic System

Currently, the United States Food and Drug Administration (US FDA) has approved three growth factors for treating H‐ARS: recombinant human granulocyte CSF (rhG‐CSF, filgrastim, Neupogen), PEGylated rhG‐CSF (pegfilgrastim, Neulasta), and recombinant human GM‐CSF (sargramostim, Leukine) [353]. Both filgrastim and pegfilgrastim act through G‐CSF receptors on neutrophil progenitor cells, stimulating neutrophil production, differentiation, and activation, while enhancing phagocytic activity and antibody‐dependent cellular cytotoxicity, thereby significantly accelerating BM recovery [353, 354]. Notably, a single dose of pegfilgrastim can increase 30‐day survival rates in nonhuman primates from 12.5 to 83% at 8 Gy and from 0 to 63% at 8.5 Gy [354]. GM‐CSF demonstrates broader hematopoietic activity than G‐CSF, targeting multiple lineages including granulocytes, monocytes, macrophages, BM‐derived dendritic cells, megakaryocytes, and erythroid progenitor cells [353, 355]. In nonhuman primate total‐body irradiation models, G‐CSF requires early administration postexposure, while GM‐CSF remains effective when given within 120 h [356]. For platelet regeneration, the thrombopoietin receptor agonist romiplostim (Nplate) improves systemic organ perfusion through platelet recovery. A single 5 mg/kg subcutaneous injection increases 60‐day survival from 32.5 to 72.5% [272, 281]. Recombinant human thrombopoietin, administered as a single 5–10 µg/kg dose 2 h postirradiation, mitigates multilineage hematopoietic damage and preserves long‐term hematopoietic function [357, 358, 359]. Nitro‐oleic acid (NO2‐OA) stimulates endogenous G‐CSF production, significantly elevating leukocyte counts and BM cellularity with a 24‐h therapeutic window [360]. These agents collectively promote hematopoietic reconstruction through direct stimulation of granulocyte and platelet lineages.

Recent investigations into drug repurposing have identified multiple molecularly targeted agents with radioprotective potential, such as N‐acetyl tryptophan glucoside [361], ACE2 agonist diminazene aceturate [362], VEGF pathway‐targeted therapies (including ACE inhibitors like lisinopril) [363], selective COX‐2 inhibitors such as meloxicam [363], TLR5 agonist entolimod [363], endothelial‐derived CCL5 [363], and others. All of them can improve the hematopoietic system injury after radiation. The prostaglandin E2 analog 16,16‐dimethyl prostaglandin E2 (dmPGE2) demonstrates dual mechanisms: reducing epithelial apoptosis via AKT/Bax regulation while protecting hematopoietic stem/progenitor cells through SIRT1 and EP3/EP4‐mediated suppression of mitochondrial/DNA damage and cellular senescence [302, 364]. Emerging strategies including microbial modulation, metabolic regulation, nanomedicine, and advanced cell therapies offer novel approaches. Fecal microbiota transplantation (FMT) from radiation‐resistant donors confers protection via microbial metabolites (short‐chain fatty acid [SCFA], tryptophan, indole‐3‐propionic acid) that enhance HSC function while attenuating inflammation [329, 330]. Polyvinylpyrrolidone‐functionalized niobium carbide (Nb2C‐PVP) MXene nanoparticles exhibit potent radical scavenging with favorable biodegradability [365]. Beyond traditional hematopoietic stem/progenitor cell transplants, engineered approaches show promise: 3D biomimetic BM microniches (HSPC/MSC cocultures) fully reconstruct hematopoiesis upon intramedullary injection [366]. PLX‐R18 placental stromal cells secrete IL‐6, IL‐8, G‐CSF, and MCP‐1 to counter hematopoietic failure [367], and MSC‐derived extracellular vesicles provide multifunctional protection through immunomodulation, antiapoptosis, and tissue regeneration [368]. These innovations address the complexity of radiation‐induced multiorgan injury while improving BM microenvironmental support, offering transformative potential for H‐ARS treatment.

#### 6.3.3 Combination Therapeutic Strategies

The intricate pathophysiology of radiation‐induced hematopoietic injury often limits the effectiveness of monotherapies, whereas combination strategies employing two or more therapeutic agents demonstrate synergistic benefits for enhanced clinical outcomes. A notable clinical example involves multicytokine therapy combining darbepoetin alfa (pegylated erythropoietin), pegfilgrastim, and romiplostim, which successfully accelerated BM microenvironment recovery in eight patients with significant radiation exposure [369]. Preclinical studies reveal that combined administration of molecularly targeted agents—specifically dmPGE2 and lisinopril—promotes megakaryocyte lineage recovery and enhances MK niche functionality in H‐ARS, leading to improved thrombocytopenia management and survival rates [363]. TCM formulations offer inherent synergistic advantages through their multicomponent nature. Hematopoietic recovery‐oriented compound decoctions, such as, DBT [370] and Guiqi Oral Liquid [371] have demonstrated efficacy in restoring BM monocytes, macrophages, and platelet counts in radiation‐ and chemotherapy‐induced myelosuppression models, while simultaneously stimulating BM stromal cell proliferation.

**FIGURE 7:**
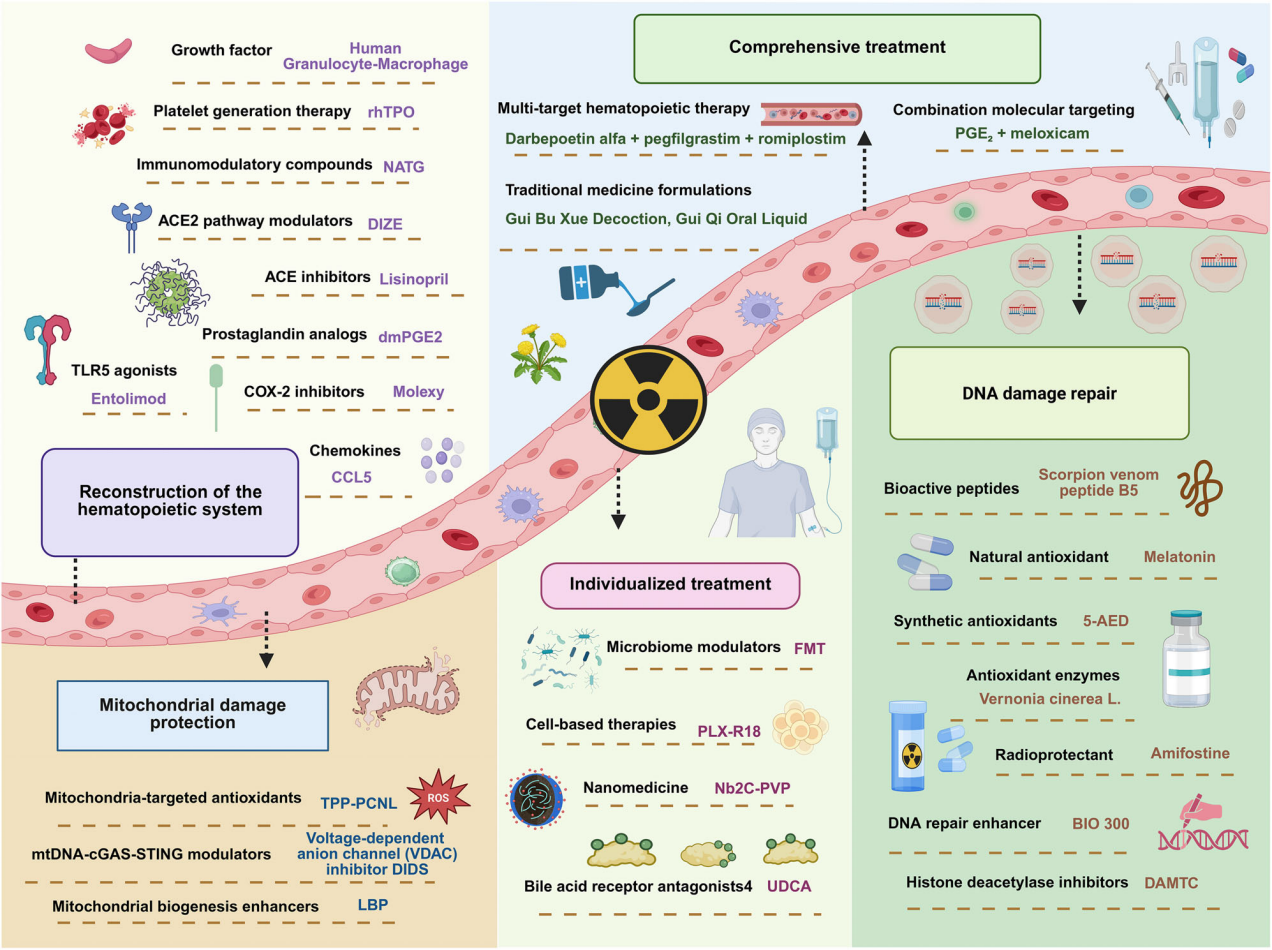
Therapeutic strategies for radiation‐induced hematological toxicity. According to the differential therapeutic objectives, therapeutic strategies for radiation‐induced hematological toxicity are principally concentrated in DNA damage repair, mitochondrial damage protection, reconstruction of the hematopoietic system, individualized treatment, and comprehensive treatment. Abbreviations: LBP: Lycium barbarum polysaccharide; UDCA: ursodeoxycholic acid.

While these findings highlight the promise of combination therapies in providing comprehensive protection against radiation‐induced hematopoietic injury, their clinical translation continues to encounter numerous challenges. Currently, US FDA‐approved growth factors (e.g., G‐CSF, GM‐CSF, romiplostim) represent the current standard of care for H‐ARS, supported by the most mature clinical data and well‐defined therapeutic windows. Their primary limitation, however, is a restricted focus on specific hematopoietic lineages, failing to comprehensively address the multisystem injuries caused by radiation exposure. Mechanistically innovative approaches, including DNA repair enhancers and mitochondria‐targeted therapies, offer novel therapeutic directions but face hurdles related to drug delivery efficiency and targeting specificity. The long‐term biosafety of associated nanomaterials also requires further evaluation. Similarly, emerging strategies, such as, microbiome modulation and cell‐based therapies, demonstrate distinct therapeutic advantages, yet their path to clinic is complicated by high manufacturing costs, complex quality control, and regulatory approval processes. Combination therapeutic strategies that integrate multiple mechanistic targets appear to represent the most promising future direction. This premise is supported by the successful clinical application of multicytokine therapies [371] and the synergistic effects of dmPGE2 combined with lisinopril in preclinical models [371]. However, optimization of dosing schedules, dose ratios, and drug–drug interactions remains a critical issue requiring urgent resolution. Furthermore, whilst TCM compound formulations demonstrate multitarget advantages, the identification of active constituents, elucidation of mechanisms of action, and establishment of quality standards require strengthening. The therapeutic paradigm for RIHT damage has evolved substantially, progressing from single‐agent approaches to integrated multilevel strategies encompassing (Figure 7): (1) molecular‐level interventions targeting DNA repair and oxidative stress; (2) cellular‐level preservation and activation of HSCs; and (3) system‐level combination therapies. As our understanding of radiation injury mechanisms deepens alongside technological advancements, future investigations should focus on: (1) optimizing drug combination regimens through systematic evaluation; (2) developing precision medicine approaches based on individual genotypic profiles and biomarker signatures; and (3) translating these findings into clinical applications for more effective radioprotection and treatment strategies.

## 7 Radiation‐Induced Intestinal Injury

Unlike organs whose functions are primarily determined by resident host cells, the intestine is a complex ecosystem maintained collectively by crypt SCs, a rapidly renewing epithelium, the mucus layer, the local immune system, the gut microbiota, and their metabolites [372]. Radiation directly damages crypt SCs and epithelial cells, disrupting the epithelial barrier and altering the ecological niches that support microbial survival. Concurrently, radiation reshapes the microbial composition and metabolite profiles, further accelerating breakdown of the intestinal barrier. Accordingly, current therapeutic approaches focus primarily on two complementary objectives: repairing the intestinal epithelial barrier and modulating the gut microbiota. Future strategies should integrate epithelial barrier reinforcement, microbiota remodeling, metabolic regulation, and anti‑inflammatory and antioxidant interventions to preserve both epithelial integrity and microbial homeostasis.

### 7.1 Epidemiology of Radiation‐Induced Intestinal Injury

The intestinal tissue is highly sensitive to IR due to its rapid cell renewal rate, making it one of the most susceptible organs to radiation damage in the body [372]. Epidemiological studies indicate that among patients receiving pelvic radiotherapy, 35–75% develop radiation‐induced intestinal injury (RIII) [373]. The incidence of RIII varies across different segments of the intestine depending on their radiosensitivity, with the descending order of susceptibility as follows: rectum, sigmoid colon, transverse colon, ileum, jejunum, and duodenum [374]. Based on disease progression, RIII can be classified into acute RIII (ARIII) and late RIII (LRIII). ARIII typically occurs during or shortly after radiotherapy (generally within 3 months), and presents with symptoms, such as, diarrhea, abdominal pain, and nausea, which usually resolves within 3 weeks after radiotherapy [375]. ARIII results from direct radiation damage to intestinal epithelial cells (IECs), characterized by massive apoptosis of crypt cells, significant villus shortening and degenerative edema, disruption of tight junction proteins, and a consequent increase in intestinal permeability [376], and breakdown of the epithelial barrier. In contrast, LRIII manifests in a delayed manner, occurring months after radiotherapy [377, 378]. It is associated with persistently increased intestinal mucosal permeability and reduced integrity of the intestinal epithelial barrier. Chromic epithelial damage promotes fibroblast deposition, vascular intimal thickening, tissue scarring, and hyalinization [379]. These pathological changes—ongoing mucosal injury, fibrosis, and vascular insufficiency—exacerbate epithelial barrier dysfunction and gut microbiota dysbiosis, ultimately leading to malabsorption and intestinal rigidity [380]. Clinically, LRIII is characterized by persistent diarrhea, malabsorption, abdominal pain, rectal bleeding, stenosis, fistula formation, and even obstruction [375, 381]. Long‐term follow‐up reveals that approximately 90% of patients experience permanent changes in bowel habits, and 30–66% of pelvic cancer survivors report chronic intestinal symptoms [378, 382, 383]. As such, RIII poses a significant threat to patients’ quality of life and overall health [384]. A deeper understanding of the common pathogenic mechanisms underlying both LRIII and ARIII is essential for optimizing clinical radiotherapy strategies and improving tumor prognosis.

### 7.2 Pathogenesis of RIII

#### 7.2.1 Multifactorial Disruption of the Epithelial Barrier

The multilayered disruption of the epithelial barrier constitues a fundamental pathological process underlying both ARIII and LRIII. This complex mechanism involves multiple interconnected components, including tight junction structural damage, cell death, vascular injury, mucus layer destruction, and immune dysregulation. Under physiological conditions, tight junction structures maintain epithelial barrier integrity by regulating paracellular permeability and preventing bacteria and antigens penetration into the lamina propria [385, 386]. Following radiotherapy, however, studies have demonstrated significant downregulation of tight junction proteins, such as, Claudin family, junctional adhesion molecule‐1, occludin, CLDN3, and ZO‐1 [387, 388], resulting in increased cell permeability and disruption of the epithelial barrier. Second, IECs undergo different pathways (including apoptosis, necroptosis, pyroptosis, immunogenic death, mitophagy, etc.) after irradiation [389, 390]. This process extends beyond directly irradiated cells, as signals from dying cells recruit adjacent nonirradiated cells into the death cascade, amplifying epithelial barrier disruption [391]. The loss of specific cell populations critically influences RIII progression. For example, Lgr5^+^ intestinal SCs, which normally sustain epithelial regeneration through self‐renewal and differentiation, undergo massive apoptosis following radiation exposure [392], severely compromising barrier repair capacity. Concurrently, vascular endothelial cells compromises microvascular integrity, enhancing vascular tone, increasing vascular permeability, and reducing blood flow [393]. This microvascular damage deprives intestinal tissue of essential oxygen and nutrients, exacerbating cellular stress and death [39]. Moreover, damaged vascular endothelial cells abnormally express adhesion molecules, including ICAM‐1, further disrupting tight junctions and inducing immune cell infiltration [385]. As mentioned above, radiation‐damaged double‐stranded DNA damage activates the cGAS/STING pathway, triggering downstream signaling cascades involving NF‐κB, MAPK, and TNF‐α, culminating in inflammatory cytokine storm. These inflammatory mediators directly compromise tight junction integrity by increasing myosin light chain kinase production while simultaneously accelerating vascular injury. Similarly, immune dysregulation further amplifies this process: aberrant activation of Th17 and Th1 cells and decreased FOXP3 expression in Tregs, promotes excessive production of IL‐1β, IFN‐γ, and T‐bet transcription factors [375, 394, 395], perpetuating inflammatory storm and aggravate the destruction of the epithelial barrier. Additionally, irradiation reduces the number of goblet cells and the expression of Mucin 2 [396, 397], compromising the protective mucus layer that normally supports mucosal barrier function. Thus, the interplay among tight junction disruption, cell death, vascular injury, mucus degradation, inflammatory responses, and immune dysregulation collectively drives epithelial barrier dysfunction and ultimately RIII pathogenesis.

#### 7.2.2 Intestinal Flora Imbalance and Metabolic Abnormalities Aggravate Epithelial Barrier Damage

**FIGURE 8:**
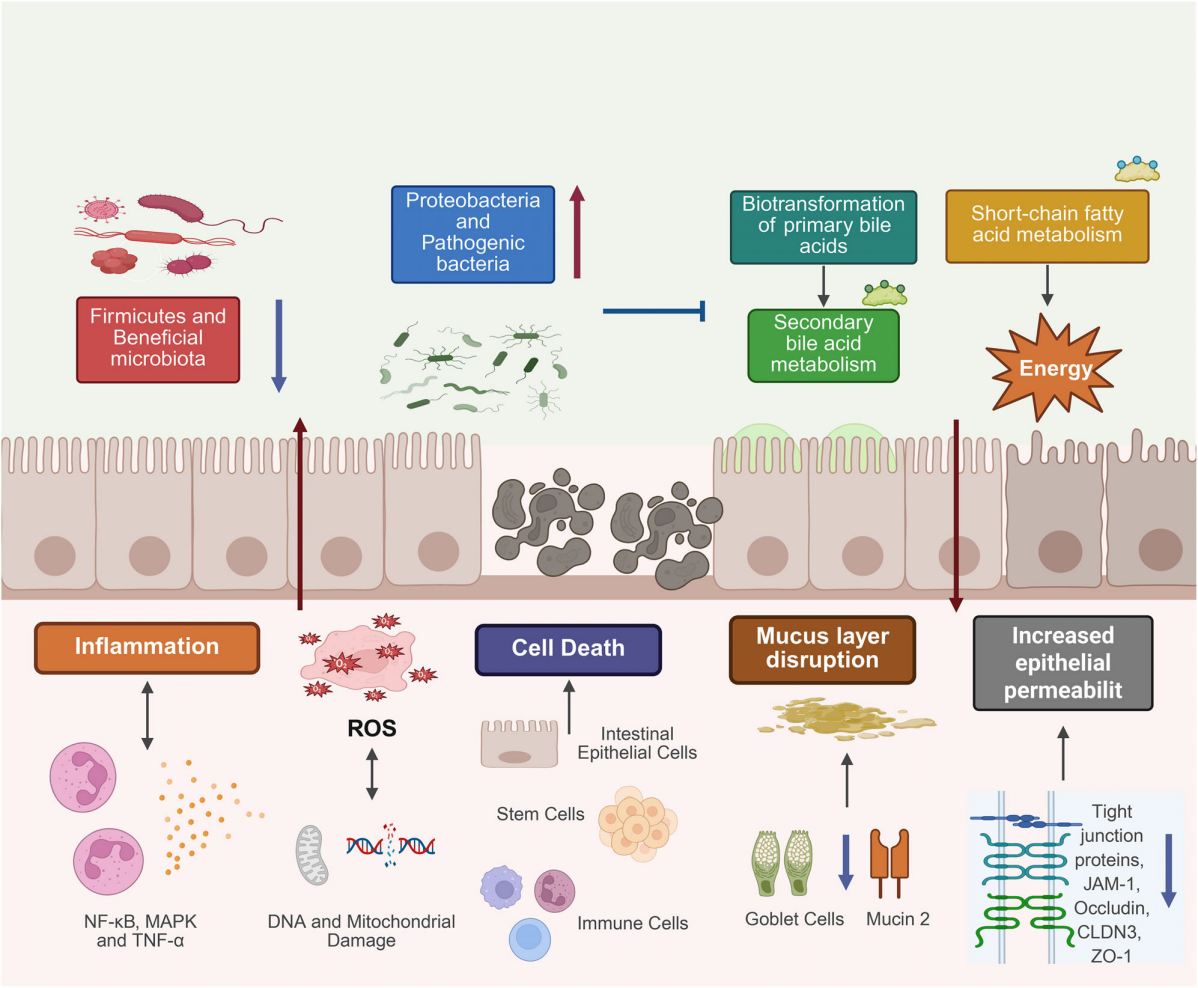
Distuption of epithelial barrier in radiation‐induced intestinal injury. Following irradiation, multiple mechanisms—including inflammation, ROS, cell death, disruption of the mucus layer, and increased cellular permeability—collectively contribute to epithelial barrier injury. Radiation exposure and the resulting intestinal epithelial barrier damages lead to alterations in intestinal microbiota composition and metabolic dysfunction, which in turn further exacerbate the injury. These two core factors, the epithelial barrier and the gut microbiota, interact reciprocally to form a vicious cycle, ultimately driving the progression and aggravation of radiation‐induced intestinal injury.

The intestinal tract harbors a unique microbial ecosystem essential for nutrient absorption and immune regulation [385]. In healthy mammals, the gut microbiota is prodominantly composed of Firmicutes and Bacteroidetes, with Proteobacteria, Verrucomicrobia, and Actinobacteria, maintaining relative stability [398, 399]. However, IR induces significant microbial dysregulation. Patients with severe chronic enteritis following cervical cancer radiotherapy exhibit markedly reduced Firmicutes abundance alongside increased Proteobacteria [386] proportions in fecal samples. Radiation exposure diminishes bacteria populations, including Bifidobacterium, impairing retinoic acid production, and subsequently reducing FOXP3 expression [400], thereby promoting inflammatory response. Clostridia, which normally facilitate regulatory T cell expression and alleviate colitis and allergic diarrhea [401], are also significantly depleted in RIII patients. Conversely, pathogenic populations expand: Shigella, a Gram‐negative facultative anaerobe, increases to about 20% relative abundance within 3 days postirradiation, while Bacteroides species proliferate rapidly by utilizing increased mucin‐derived oligosaccharides [376]. Lipopolysaccharide (LPS) produced by expanded Bacteroides populations abnormally activates intestinal immune cells, exacerbating local inflammation. LPS also directly disrupts epithelial tight junctions, increases intestinal permeability, and promotes endotoxin translocation, which leads to epithelial barrier dysfunction [402]. Beyond the imbalance of bacterial abundance, radiation‐induced metabolic abnormality in intestinal flora critically contribute to barrier damage. Second, bile acids normally exert direct antimicrobial effects by embedding their hydrophobic ends in the cell membrane of Gram‐positive bacteria [403] and serve as signaling molecules activating host nuclear receptors, such as, GPBAR1(TGR5), thereby modulating microbiota composition and immune homeostasis [404]. Following irradiation, reduced Firmicutes abundance and pathogenic bacterial expansion inhibit primary bile acid bioconversion, resulting in significantly decrease secondary bile acids. SCFA metabolism is similarly compromised. SCFA levels decline sharply within 24 h postirradiation, with propionic acid, isobutyric acid, isovaleric acid, butyric acid, and caproic acid all significantly reduced by 3 days [376]. Radiation‐induced SCFAs depletion impairs FFAR2/3 and GPR109A‐mediated hormone secretion, epigenetic modification, and immune regulation [405, 406], while also reducing GPR43‐mediated energy supply to enterocytes (butyrate meets about 70% of the energy demand of colonic cells [405]. Furthermore, SCFA depletion facilitates LPS translocation and activates inflammatory signaling pathways [376], exacerbating intestinal epithelial barrier dysfunction [407]. In summary, the core pathological mechanism of RIII involves a self‐perpetuating vicious cycle wherein epithelial barrier dysfunction and gut microbiota imbalance mutually reinforce each other (Figure 8). This understanding underscores that targeted barrier repair and microbiota reconstruction represent key therapeutic strategies for clinical RIII management.

### 7.3 Prevention and Treatments for RIII

For RIII, prevention is centered on minimizing intestinal radiation exposure, physically increasing the distance between the target and bowel when feasible, and preserving the epithelial barrier–microbiota axis before dysbiosis and chronic permeability changes occur. Barrier‐protective nutrients, anti‐inflammatory and antioxidant agents, probiotics, microbial metabolites, and spacer‐based approaches should therefore be considered within a preventive continuum before being discussed as treatments for established intestinal injury.

#### 7.3.1 Strategies for Intestinal Epithelial Barrier Repair

Current clinical therapeutic strategies targeting intestinal epithelial barrier repair in RIII typically employ multiple targets, such as, restoring the expression of tight junction proteins, promoting cell proliferation, and reducing inflammation and oxidative stress. For example, prophylactic administration of TLR4 inhibitors or NF‐κB pathway blockers, combined with antioxidant nutrients (vitamins C and E) and ω‐3 polyunsaturated fatty acids, can attenuate LPS‐induced TLR4/MyD88/NF‐κB pathway activation while targeting both inflammation and oxidative stress [376]. Ergothionein [408], Leyamipide [409, 410], OA‐rich enteral nutrition [384], and baicalein [411] can promote the expression of tight protein junction, reduce cell apoptosis, promote cell proliferation and help the recovery of intestinal epithelial barrier. While crocetin [412], dexmedetomidine [413], resveratrol [385], pravastatin [414], and mitochondrial transfer [415]. Because of its antioxidant, anti‐inflammatory, and antiapoptotic properties, it has become a candidate for intestinal protective therapy [413]. Nanoengineering drugs are also representative of RIII multitarget therapeutic strategies, such as, CS/PEEC‐AMF NPs [416] and MSC‐derived extracellular vesicles (MSC‐EVs) [417], which have been confirmed to have various effects, such as, anti‐inflammatory, antioxidation, promoting cell regeneration, inhibiting cell apoptosis, and upregulating the expression of tight junction proteins. Single‐target drugs, such as, isoglycyrrhizin [418], baicalin [411], statins (article 56), and so on, can repair the intestinal epithelial barrier by restoring the expression of tight junction proteins. Others, such as, mevalonate pathway inhibitors, can preserve endothelial function and improve radiation‐induced tight junction dysfunction by preserving the KLF2‐TM/eNOS axis [419]. Cell regenerating‐promoting drugs (Apelin [420], luteolin, total flavonoids of castanea roxobin leaf [421], quercetin 3‐O‐rutin [422], ghrelin [392], dimephosphon [423], and others) have also been confirmed to promote intestinal cell proliferation and repair the damaged epithelial barrier through Wnt/Notch/EGF signaling pathways. MSCs treatment also showed a good effect on epithelial barrier regeneration. Studies have shown that MSCs promote Lgr5^+^ ISCs growth by increasing Wnt/β‐catenin signaling pathway activation and secrete IL‐6 to promote epithelial regeneration [424, 425]. Ginsenoside Rg3 can inhibit the TLR4/MyD88/NF‐κB pathway, downregulate the expression of IL‐1β and upregulate the expression of IL‐10, thereby alleviating acute radiation proctitis [386]. In addition, ultra‐high dose‐rate radiotherapy (FLASH radiotherapy) can also reduce the loss of crypt proliferative cells and preserve regenerative cryphs, and the 90‐day survival rate after total abdominal irradiation is significantly increased, with a greater number of intestinal cryphs and greater villus height [426]. Notably, Tregs play a key role in intestinal tissue repair by enhancing barrier function and promoting epithelial SC renewal. FOXP3^+^ Tregs and the resident GATA3^+^Helios^+^ Treg population can help epithelial SCs regeneration by secreting effector cytokines [394] (such as, IL‐10), which is a potential therapeutic direction for RIII.

#### 7.3.2 Strategies for Regulating Intestinal Flora

Advances in metagenomics, metabolomics, and multiomics integration have substantially elucidated the critical role of gut microbiota and their metabolites in maintaining intestinal barrier integrity and systemic immune homeostasis [427, 428]. Oral administration of multiple strains of probiotics can effectively alleviate radiation‐related tissue damage, significantly reduce intestinal epithelial damage, and partially restore intestinal microbiota diversity [429]. A clinical study involving 54 cervical cancer patients demonstrated that probiotic supplementation during radiotherapy reduced diarrhea incidence from 82.1 to 53.8% [430]. Lactobacillus rhamnosus GG promotes epithelial cell regeneration through TLR2, MyD88, and AK2/STAT3 signaling pathways while modulating Th17/Treg balance [431] and alleviating oxidative stress and inflammation. These properties position it as a promising radioprotective agent. Metformin has similarly demonstrated efficacy in improving gut microbiota composition and diversity. Patients receiving metformin during abdominal radiotherapy had a significant increase in the number of Lactobacillus in the gut during abdominal radiotherapy, accompanied by a decrease in the duration of diarrhea [396, 432]. Since the first report of successful treatment of radiation‐induced diarrhea with FMT in 2023 [433], a number of studies have confirmed that FMT can restore beneficial bacteria, such as, Lactobacillus and Triclospirillaceae, regulate the expression of tryptophan and other metabolites, and participate in damage repair [434]. The improvement rate of radiation enteritis symptoms within 3 months reached 77.3% [435]. It provides an innovative treatment approach for RIII starting from the metabolites of bacterial flora. At present, the metabolites of microbiota used for RIII treatment mainly include SCFA tryptophan metabolites and bile acids. As for SCFAs, butyrate enema significantly alleviated RIII in the abdominal and pelvic region by regulating GPR41/43 receptors [436]. Both propionate and acetate can alleviate RIII by regulating the number and function of immune cells. Valeric acid, on the other hand, is associated with the mechanical stability of cells [436] and alleviates RIII by upregulating the expression of cytoskeleton‐related proteins, such as, Keratin 1 (KRT1), through the AML1/KRT1 signaling pathway. Tryptophan metabolites (indole‐3‐propionic acid, indole‐3‐formaldehyde, 3,3′‐diindole‐methane, indole‐3‐lactic acid, and kynouric acid) mainly play a role in reducing oxidative stress, protecting normal epithelial cells, and maintaining the proliferation and differentiation of intestinal SCs [436, 437, 438]. Lithocholic acid and allolithocholic acid belong to bile acid metabolites. Among them, lithocholic acid protects against RIII by upregulating the expression of TGR5 and YAP1 in intestinal crypts [439]. Isolicholic acid, on the other hand, mediates the increase of histone H3 lysine 27 acetylation (H3K27ac) at the FOXP3 locus, thereby increasing the expression of FOXP3 in Tregs at the transcriptional level and exerting a protective effect [440]. In addition, LPS can also mediate radioprotection through activation of TLR4 receptor [436]. Based on the molecular mechanism of intestinal flora and its derived products mediating radioprotection through a variety of signaling pathways, clinical intervention can consider the synergistic effect of exogenous flora regulation and endogenous metabolic activation: specific probiotics or FMT can restore intestinal microecological balance; combined with dietary interventions, such as, high‐fiber diet to promote SCFAs production and tryptophan supplementation to enhance indole metabolite synthesis, to ensure the continuous supply of endogenous protective substances. It is believed that this combined strategy may be more effective in maintaining intestinal homeostasis during radiotherapy [441].

Due to the particularity of intestinal flora, the therapeutic administration of RIII is different from that of other organs. In the future, the clinical treatment of RIII can integrate the following four aspects to establish combined intervention strategies. The first is epithelial barrier strengthening. The epithelial barrier repair drugs can be given before and after irradiation to enhance the expression of tight junction proteins and promote the proliferation of epithelial cells. The second is gut microbiota remodeling with combination probiotics or fecal transplantation, with limited use of broad‐spectrum antibiotics. The third is to regulate intestinal metabolism through supplementation of essential amino acids, bile acids, SCFAs, drugs, or diet. Finally, anti‐inflammatory drugs are used in combination with antioxidant nutrients (vitamins C and E) to reduce inflammatory response and oxidative stress damage. This combined strategy aims to maintain the intestinal epithelial barrier and microbiota balance in all aspects, thereby minimizing RIII.

## 8 Other Clinically Significant Radiation‐Induced Injuries

### 8.1 Radiation‐Induced Oral Mucositis

Radiation‐induced oral injury occurs predominantly in patients receiving radiotherapy for head and neck cancers and manifests as persistent oral mucositis characterized mainly by erythema, erosion, and ulceration of the oral mucosa, with severe cases accounting for up to 52% of patients [442]. Owing to the specific features of the oral microenvironment, the pathological mechanisms underlying radiation‐induced oral injury resemble those of RIII, with both resulting from the combined effects of barrier disruption and microbial dysbiosis. During the acute phase of radiation‐induced oral injury, pain typically develops in the first week of radiotherapy, ulcers emerge in the second week, and these may coalesce into extensive mucosal ulcerations by the third week. Persistent ulcerative mucositis may last for 2–4 weeks after the final radiation dose [443]. IR causes marked thinning of the oral mucosa, primarily through the depletion of epithelial cells, particularly basal cells. Notably, lipid peroxidation‐driven ferroptosis, rather than apoptosis alone, was identified as a principal mechanism of radiation‐induced basal epithelial cell death [444, 445, 446]. During the early stage, fibroblasts provide metabolic support to basal cells through hypoxia‐inducible factor 1α‐ODC1‐mediated polyamine synthesis. At later stages, however, fibroblast depletion and senescence, together with polyamine exhaustion, result in decompensation of this protective mechanism. These alterations, compounded by lipid peroxidation‐driven ferroptosis, collectively exacerbate the collapse of both the physical and immune barriers [445, 446]. Concurrently, oral microbial diversity decreases, with reductions in health‐associated taxa, such as, Streptococcus and increases in pathogenic Gram‐negative bacteria, anaerobes, and other microorganisms, accompanied by enhanced biofilm formation, bacterial virulence, and antibiotic resistance [447]. Microbial dysbiosis further aggravates barrier injury, activates inflammation‐associated pathways, such as, NF‐κB and the NLRP3 inflammasome, and promotes the release of inflammatory mediators including IL‐1β, IL‐6, IL‐18, and TNF‐α, thereby converting barrier disruption into persistent oral mucosal inflammation [448].

Therapeutic strategies for radiation‐induced oral mucositis include both pharmacological and nonpharmacological interventions, encompassing early prevention, restoration of microbial homeostasis, mucosal protection and regeneration, pain and infection control, and nutritional support; however, no universally accepted optimal regimen has yet been established [448, 449]. Probiotic‐mediated modulation of the oral microbiota represents a promising pharmacological strategy for the treatment of radiation‐induced oral mucositis. Lactobacillus brevis CD2 lozenges and Bacillus clausii UBBC07 may restore microbial homeostasis, preserve the structural integrity of the mucosal barrier, and modulate the local immune microenvironment [450]. However, clinical efficacy may vary substantially according to the probiotic strain, formulation, dose, timing and duration of administration, patient‐selection criteria, radiation dose, and chemoradiotherapy regimen. Probiotics should be used cautiously in immunocompromised or neutropenic patients because of potential safety concerns, including horizontal gene transfer, bacterial translocation, recurrent infection, and sepsis [451, 452, 453]. Antimicrobial agents, such as, iseganan and amphotericin B, can effectively suppress pathogenic microorganisms, but their prolonged or isolated use may exacerbate microbial dysbiosis, and their optimal clinical application remains controversial [449]. Agents that promote mucosal repair represent the most diverse therapeutic category and include antioxidants, anti‑inflammatory agents, nutritional supplements, immunomodulators, biological agents that promote epithelial proliferation and tissue regeneration (such as, colony‑stimulating factors and MSCs), and barrier‑protective and moisturizing formulations, notably hyaluronic acid and artificial saliva [449, 454]. These agents can ameliorate mucosal injury through antioxidant, anti‐inflammatory, antimicrobial, and nutritional effects, as well as by promoting epithelial repair. Although they have produced encouraging therapeutic outcomes in clinical practice, several unresolved issues require further investigation. For example, although MSCs have been shown to improve epithelial height, mucosal thickness, and radiation tolerance, the available evidence remains confined to animal studies, with insufficient supporting clinical data [455, 456]. Low concentrations of spermidine or spermine can protect basal cells and preserve mucosal integrity; however, their effects are biphasic and concentration‐dependent, raising safety concerns regarding long‐term administration [457]. Natural products exhibit antimicrobial, antifungal, anti‐inflammatory, and antioxidant activities while promoting epithelialization; however, the existing evidence is highly heterogeneous, and potential adverse effects—including nausea, allergic reactions, dental caries, and drug interactions—require careful consideration [443]. Nonpharmacological strategies primarily aim to reduce radiation exposure by decreasing both the delivered dose and the irradiated volume, using tongue‐displacement stents and adopting proton therapy. Standardized oral cleansing and moisturizing, maxillofacial and intraoral massage, symptom monitoring, and individualized health education are also clinically implemented to alleviate radiation‐induced oral mucosal injury [449]. However, network meta‐analyses indicate that nonpharmacological interventions alone have limited efficacy in severe cases. Therefore, an integrated strategy combining pharmacological and nonpharmacological interventions should be adopted, with continuous provision of basic oral care, moisturization, nutritional support, and health education. Depending on individual risk and disease course, these measures should be complemented by appropriate pharmacological interventions targeting microbial dysbiosis, oxidative stress/inflammation, mucosal regeneration, pain, and infection, thereby simultaneously correcting microbial imbalance and barrier disruption while ensuring treatment effectiveness, stability, and safety.

### 8.2 Radiation‐Induced Nephropathy

The pathological progression of radiation‑induced kidney injury is shaped by temporal dynamics, renal structural heterogeneity, and irradiation modality. In addition to conventional interventions—antioxidants, DNA damage‑repair agents, and radiation‐dose reduction—inhibition of renal tubular reabsorption and modulation of the renin–angiotensin system represent promising therapeutic approaches. Radiation‐induced kidney injury exhibits a pronounced time‐dependent course and is generally clinically silent during its early stages. At 6–18 months after radiotherapy, patients may develop proteinuria, hypertension, azotemia, and persistent anemia, which can subsequently progress to uremia and chronic renal failure; severe cases may ultimately require dialysis or kidney transplantation [457, 458]. Moreover, as one of the most radiosensitive abdominal organs, the kidney is particularly vulnerable to radiation owing to its complex structural organization. Following exposure to IR, the glomeruli and renal tubules are the most severely affected compartments [458, 459]. Within the glomerulus, radiation‐induced cytoskeletal remodeling and foot‐process effacement in podocytes disrupt the filtration barrier and promote proteinuria [460, 461]. Concurrently, DNA damage in glomerular endothelial cells, mitochondrial ROS generation, and activation of the TGF‐β/Smad pathway collectively drive glomerulosclerosis and interstitial fibrosis, eventually compromising glomerular filtration in severe cases [459]. Tubular injury is characterized primarily by epithelial necrosis, vacuolar degeneration, luminal dilatation, cast formation, and apoptosis, which impair tubular reabsorptive capacity and ultimately lead to electrolyte disturbances and renal dysfunction [462, 463].

In addition to its temporal and structural determinants, the irradiation modality critically influences the pathological progression of radiation‐induced kidney injury. Studies have shown that, per unit of absorbed dose, radionuclide therapy produces three to sevenfold greater biological damage than external‐beam radiotherapy [464]. This difference is largely attributable to the glomerular filtration of small‐molecule radiopharmaceuticals, followed by their reabsorption and retention in the proximal tubules, resulting in concentrated injury to the renal tubules and the outer stripe of the outer medulla. Depending on the radiation range, radionuclides may also cross‐irradiate adjacent glomerular regions, thereby exacerbating renal damage [465]. For example, the β‐particles emitted by ^90^Y have higher energy and a longer tissue range than those emitted by ^177^Lu, enabling radiation to extend from tubular binding sites to more distant glomeruli. Consequently, the risk of severe nephrotoxicity can reach 33.6%, substantially exceeding that associated with ^177^Lu [466].

Reducing the radiation dose absorbed by the kidneys remains the primary strategy for preventing and mitigating radiation‑induced kidney injury. CT‑guided interstitial high‑dose‑rate brachytherapy enables focal single‑fraction irradiation using a ^1^ ^9^ ^2^Ir source and may spare healthy renal tissue by minimizing respiratory‑motion‑related exposure. Stereotactic body radiotherapy likewise offers noninvasive, fractionated treatment with favorable dose distribution and the potential to reduce radiation damage [467]. For nephrotoxicity induced by radionuclide therapy, pharmacological inhibition of tubular reabsorption may be considered in conjunction with optimization of radionuclide selection, dose adjustment, fractionated administration, and promotion of urinary excretion. Evidence indicates that concurrent infusion of L‑arginine or L‑lysine competitively inhibits proximal tubular reabsorption, reducing the renal radiation dose by 9–53% [468]. The combination of succinylated gelatin and lysine can similarly reduce renal tracer uptake by 62% [469]. Furthermore, introducing a cleavable linker between a radiometal–chelator complex and a peptide can reduce renal uptake without compromising tumor uptake [470], thereby limiting kidney injury.

As with other forms of radiation injury, pharmacological interventions primarily involve antioxidants [471], DNA damage‑repair agents [458], and strategies that suppress apoptosis [472]. With the recent expansion of research into metabolic regulation, targeting lipid metabolism has also yielded encouraging results in radiation‑induced kidney injury. Rosuvastatin inhibits cholesterol synthesis, activates SIRT1/FOXO3a signaling and PINK1/Parkin‑mediated mitophagy, and restores redox homeostasis, thereby improving renal function and histopathological injury after both acute and fractionated irradiation [472]. Combined pretreatment with zoledronic acid and pravastatin enhances the repair of DNA double‑strand breaks. Meanwhile, preservation of SMPDL3b expression, nuclear sphingolipid metabolism, and nuclear membrane fluidity may represent additional therapeutic avenues, although the underlying mechanisms require further clarification [458]. Nevertheless, as lipid metabolic regulation remains an emerging field, most candidate agents have thus far been evaluated only in animal models, and further research is required to establish appropriate dosing regimens for clinical application.

Angiotensin‐converting enzyme inhibitors (ACEIs) and Angiotensin II receptor blockers (ARBs), which suppress adverse tissue responses mediated by the renin–angiotensin–aldosterone system, have long been prominent therapeutic targets in kidney disease. In radiation‐induced kidney injury, ACEIs and ARBs likewise represent major preventive and therapeutic agents [459]. ARBs combined with lysine can attenuate nephrotoxicity following high‐activity ^177^Lu therapy [473]. However, the effects of ACEIs and ARBs are dose dependent, and their therapeutic efficacy against established injury remains inconsistent [459, 474]. Collectively, these findings underscore the need for further mechanistic investigations and optimization of therapeutic regimens for radiation‐induced kidney injury.

### 8.3 Radiation‐Induced Brain Injury and Cognitive Decline

Based on the timing of clinical symptom onset, radiation‐induced brain injury can be classified into acute, early‐delayed and late‐delayed phases [475]. Acute injury develops within hours to days after irradiation and is characterized primarily by cerebral edema, disruption of the blood–brain barrier (BBB) and vascular abnormalities [476]. Early‐delayed injury occurs several weeks to months after irradiation and is associated with reversible demyelination, secondary BBB disruption, and cognitive impairment [477, 478]. Late‐delayed injury emerges between 6 months and several years after irradiation and involves pronounced vascular alterations and glial cell damage, ultimately leading to extensive lesions in both white and grey matter, cerebral hypoperfusion and cognitive impairment of varying severity [477]. Acute and early‐delayed injuries are largely reversible, whereas late injury is typically persistent and irreversible [479].

Glial cells are essential structural and functional components of the brain, and distinct glial populations play markedly different roles during the progression of radiation‐induced injury. Under physiological conditions, astrocytes perform multiple neuroprotective functions, including metabolic support, axonal guidance, synaptic transmission, and regulation of the BBB. Following irradiation of brain tissue, astrocytes are rapidly activated and undergo proliferation and hypertrophy, thereby contributing to the repair of damaged tissue [480, 481]. As injury persists, reactive astrocytes exhibit increased expression of glial fibrillary acidic protein, enhanced secretion of proinflammatory mediators and vascular endothelial growth factor, and may ultimately contribute to glial scar formation [482]. Oligodendrocytes account for approximately 45% of all glial cells and constitute the glial population most sensitive to radiation during the acute phase [478, 482]. Evidence indicates that irradiation impairs the capacity of oligodendrocyte precursor cells to differentiate into astrocytes and neurons [483]. Concurrently, radiation can directly induce apoptosis in mature oligodendrocytes, resulting in demyelination‐associated neuroinflammation, which represents a major pathological basis of delayed radiation‐induced brain injury [482]. Microglia mediate immune surveillance, inflammatory regulation, and neural repair, and their transient activation facilitates the acute response to injury. Following irradiation, microglial activation shifts from the anti‐inflammatory M2 phenotype toward the proinflammatory M1 phenotype, whereas sustained activation promotes chronic neuroinflammation and delayed cognitive deficits [484, 485, 486]. As discussed above, irradiation can also disrupt the intestinal microbiota. Recent studies have introduced the concept of the gut–brain axis, through which intestinal microorganisms and their metabolites interact with the immune system and influence the central nervous system predominantly via microglia [482]. Accordingly, radiation‐induced dysbiosis may trigger systemic inflammation and microglial activation within the brain, ultimately contributing to cognitive decline [487]. Moreover, cranial radiotherapy compromises BBB integrity, further increasing the likelihood that the intestinal microbiota and its metabolites can influence the central nervous system. Nevertheless, current evidence is derived predominantly from mouse models, and the precise mechanisms and clinical relevance of the gut–brain axis in radiation‐induced brain injury remain to be established [482].

Beyond technological optimization, dose control, anti‐inflammatory interventions, and antioxidant approaches commonly used to mitigate radiation‐induced injury, current therapeutic strategies for radiation‐induced brain injury primarily focus on controlling cerebral edema, protecting neural tissue and promoting injury repair, and modulating the gut–brain axis [487]. For edema control, glucocorticoids remain the first‐line treatment for radiation‐induced brain injury and can rapidly alleviate symptoms by reducing cerebral edema and inflammation; however, prolonged administration may lead to steroid dependence, gastrointestinal bleeding, and infection [488]. Other antiedema treatments also have limitations: recurrence may occur after discontinuation of bevacizumab, whereas clinical evidence supporting Boswellia serrata remains limited, highlighting the need for further investigation [489, 490]. Therapeutic strategies for neuroprotection and neural repair are comparatively diverse. Established approaches for neuroprotection and cognitive rehabilitation, including hyperbaric oxygen therapy, memantine, and donepezil, have been investigated or clinically evaluated [487]. SCs have emerged as a major focus of recent research, encompassing exosomes, other extracellular vesicles and growth factors [487, 491]. Microbiota‐restorative strategies targeting the gut–brain axis, similar to those described for RIII, can improve cognitive performance following cranial irradiation. However, the available evidence remains largely dependent on mouse models, and the optimal microbial strains, doses, therapeutic windows, and long‐term consequences have yet to be defined [492]. Notably, brain–computer interface technology has been proposed as a means of directly modulating neural‐circuit function, enhancing signal transmission and restoring cognitive performance without interfering with tumor control or relying on ambiguous biomarkers or heterogeneous molecular pathways. However, no direct experimental or clinical evidence currently supports its use in RIBI, and this potential is extrapolated largely from other neurological disorders and basic research [478]. In adults with cancer, nonpharmacological interventions—including mindfulness‐based interventions, yoga, relaxation techniques, music therapy, acupuncture, tai chi or qigong, reflexology, and aromatherapy—may alleviate anxiety and depressive symptoms during or after treatment and support emotional well‐being and quality of life [493].

## 9 Commonalities and Cross‐Organ Perspectives in Radiation‐Induced Toxicity

### 9.1 Shared Mechanisms: Inflammation, Fibrosis, and Vascular Damage

Despite marked differences in tissue architecture and radiosensitivity among the heart, lungs, skin, hematopoietic system, gastrointestinal tract, brain, kidneys, and oral cavity, radiation‑induced injuries across these organs share a common pathological trajectory. This process is characterized by oxidative stress, DNA damage, mitochondrial dysfunction, immune dysregulation, and extensive cellular senescence and death, ultimately progressing toward vascular damage, inflammatory responses, and fibrosis.

Inflammation represents a central driver of radiation‐induced pathological progression and is primarily regulated through inflammatory signaling pathways and inflammasomes. TGF‐β signaling constitutes a major regulatory axis that activates inflammatory responses, promotes fibrosis, and upregulates multiple forms of PCD. Key components of this interconnected signaling network—including nuclear factor‐κB, p53, NRF2, mitogen‐activated protein kinase, and phosphoinositide 3‐kinase/protein kinase B—together with their downstream effectors, are extensively involved in the pathological progression of radiation injury across affected organs [70, 85]. The NLRP3 inflammasome constitutes another pivotal regulatory axis that sustains inflammatory responses through multiple mechanisms. Following IR exposure, DAMPs and damaged mitochondria cooperatively activate NLRP3, resulting in several downstream effects: (1) initiation of the IL‐1β/IL‐6/CRP signaling cascade and consequent induction of inflammatory responses; (2) promotion of gasdermin D cleavage, thereby triggering pyroptosis; (3) stimulation of MMP release, leading to ECM degradation and facilitating the infiltration of diverse immune‐cell populations; and (4) increased production of reactive oxygen and nitrogen species, which further exacerbates oxidative stress [72, 73, 74, 75].

Persistent inflammation drives the transition from acute injury to chronic fibrosis. Signaling pathways downstream of the inflammatory mediator TGF‐β promote the transdifferentiation of fibroblasts into myofibroblasts [230], accompanied by excessive α‐SMA production and collagen deposition [232], and ultimately induce extensive ECM remodeling [233]. In addition to the central role of TGF‐β, radiation itself activates both tissue‐resident fibroblasts and BM‐derived fibroblasts and generates new mesenchymal‐like cells through EMT and EndMT, thereby expanding the myofibroblast population [232, 233, 237]. Cellular senescence, immune‐cell infiltration and radiation‐induced phenotypic alterations, as described above, further sustain fibroblast activation and ultimately culminate in tissue fibrosis. Radiation‐induced vascular injury serves both as an initiating factor for chronic inflammation and as a catalyst for fibrosis. Radiation‐induced DNA damage and mitochondrial dysfunction cause extensive vascular endothelial‐cell death and the release of inflammatory mediators, such as, HMGB1, IL‐1α, and IL‐33. Radiation also disrupts interendothelial adhesion, compromises microvascular integrity, increases vascular tone, and enhances vascular permeability [311, 312], thereby establishing a proinflammatory microenvironment [67]. Moreover, damaged vascular endothelial cells aberrantly express molecules, such as, ICAM‐1, which promote immune‐cell infiltration [385] and subsequently facilitate fibroblast transformation. This unified pathogenic framework of radiation injury identifies new therapeutic targets for broad‐spectrum radioprotective strategies and provides an important theoretical foundation for the development of novel integrated therapeutic approaches.

### 9.2 Comparative Efficacy of Radioprotectors Versus Mitigators

The principal distinctions between radioprotectors and radiation‐injury mitigators lie in their therapeutic objectives and timing of intervention. Radioprotectors are typically administered before or during irradiation, with the primary aims of scavenging ROS, enhancing DNA repair, protecting mitochondria, and preserving vascular endothelial integrity, thereby intercepting the early stages of tissue injury. Amifostine is currently the only radioprotector approved by the US FDA. When administered before radiotherapy, it exerts protective effects by scavenging free radicals, preserving mitochondrial function and maintaining vascular endothelial integrity, although its clinical use is associated with adverse effects, such as, hypotension. In addition to amifostine, antioxidants constitute the most extensively investigated class of radioprotectors and include vitamins, natural medicines, and their bioactive constituents. However, most radioprotective agents have been evaluated only in preclinical animal studies and must be administered before or immediately after irradiation to achieve efficacy. Their narrow therapeutic window and associated adverse effects therefore substantially constrain clinical translation [7].

By contrast, radiation‐injury mitigators are primarily administered after irradiation or after tissue injury has become established. They attenuate radiation injury and delay disease progression by suppressing persistent inflammation, alleviating fibrosis, and restoring cellular and tissue function. Compared with radioprotectors, radiation‐injury mitigators encompass a broader range of agents, act through diverse mechanisms, and exhibit pronounced organ specificity. Drug class, dose, and route of administration must therefore be selected according to the affected organ and the clinical manifestations of radiation injury. As discussed above, the efficacy of single‐target agents can also be limited by compensatory interactions among inflammatory, programmed cell‐death, and fibrotic pathways. Precision‐delivery strategies‐based predominantly on nanocarriers provide a feasible platform for multitarget combination therapy, although dose optimization, synergistic toxicity, delivery efficiency, and long‐term safety remain to be addressed [209]. Emerging evidence further indicates that several antioxidants—including melatonin, resveratrol, curcumin, hesperidin, and tanshinones—as well as probiotics can function as both radioprotectors and radiation‐injury mitigators because of their diverse mechanisms of action, highlighting their considerable potential for the prevention and treatment of radiation injury. Nevertheless, most existing studies remain preclinical and have examined these two roles separately. Further investigation is therefore required to establish clinical strategies that exploit these agents concurrently as radioprotectors and radiation‐injury mitigators.

### 9.3 Toward Organ‐Specific Dose Constraints and Predictive Models

Current organ‐specific dose‐constraint standards are derived primarily from predictions generated using radiation‐injury models. As discussed in Section 2, the clinical manifestations of radiation injury exhibit characteristic dose dependence and organ specificity and are influenced by multiple modifying factors. Dose constraints may differ even among individual substructures within the same organ, as exemplified by the heart. A retrospective analysis of 122 patients with esophageal cancer treated with definitive RT or chemo‐RT showed that LA V15 was the only parameter that remained significantly associated with Grade ≥3 major adverse cardiac events (MACE). Receiver operating characteristic curve analysis identified optimal LA V15 thresholds of 76.5 cc and 97.6 cc for Grade ≥3 and Grade ≥4 MACE, respectively [494]. In a separate cohort of 475 patients with NSCLC, Johnson‐Hart et al. identified the mean dose to the aortic and coronary arterial regions as predictive of survival and used 16.2 Gy as a conservative threshold [495].

Accordingly, radiation‐injury prediction models must integrate total dose, mean organ dose, dose–volume parameters, fractionation schedules, radiation‐field size, organ substructures, and the spatial relationships among organs at risk to effectively estimate dose constraints and injury progression. Using adaptive contouring in Monaco, Wang et al. defined the “oropharyngeal mucosa” as a 2‐mm expansion of the oropharyngeal air cavity. Compared with constraining the oropharynx alone, additionally constraining the mucosa reduced the mean doses to the mucosa and oropharynx from 4057.84 to 3378.76 cGy and from 4318.34 to 3784.29 cGy, respectively (both *p* < 0.0001) [496]. In addition to multivariable approaches, the integrated assessment of physical and biophysical parameters, biological indicators, and clinical variables is now broadly regarded as essential for evaluating radiation dose and the resulting biological injury [497].

Among physical parameters, radiomics has attracted increasing attention in recent years. The integration of machine learning, deep learning, and multimodal imaging can improve the identification and delineation accuracy of the sinoatrial node, atrioventricular node, and other cardiac substructures, thereby facilitating dosimetric studies of the cardiac conduction system. Nevertheless, interindividual variability, organ complexity, differences among imaging techniques, and variations in clinician expertise pose substantial challenges to the development of image‐based assessment models [498]. Biomarker candidates are similarly diverse and include genes, proteins, and metabolites. Common biomarkers include FDXR, DDB2, CDKN1A, PCNA, BAX, and GADD45, which are genes associated with TP53 or NF‐κB signaling pathways and are predominantly measured in peripheral blood [497]. However, the abundance of radiation–responsive genes may exhibit either linear or linear‐quadratic relationships with radiation dose within the range of 0–4 Gy, whereas these responses tend to reach saturation at higher doses and may vary among laboratories [499]. Consequently, combining multiple biomarkers is more likely to enable the prediction of radiation dose, the systemic or localized extent of injury, injury severity, and potential therapeutic approaches [500]. Overall, to establish more rational organ‐specific dose constraints, future prediction models should incorporate large‐sample prospective designs, standardized delineation protocols, advanced imaging approaches, AI, and multimarker combinations [498, 500].

## 10 Conclusion and Perspectives

This review systematically examines the pathological process, molecular mechanisms, and current treatment strategies for radiation‐induced tissue injuries. Among the diverse therapeutic strategies reviewed, precision drug delivery systems based on novel materials and technologies hold the most transformative clinical potential. Nanomedicine platforms enable spatiotemporal control of drug release through integrated targeting, responsiveness, and multifunctionality. Additionally, 3D bioprinting technology opens new frontiers in tissue engineering, from assessing radiation damage with 3D‐printed fibroblast constructs to fabricating autologous cell‐based wound repair materials for accelerated healing—all customizable bioinks permitting patient‐specific solutions. More importantly, precision drug delivery systems preserve the multitarget synergy of combination therapies while enhancing tissue specificity and therapeutic efficacy, simultaneously addressing off‐target effects and biocompatibility issues. Integrating such delivery systems with multitarget interventions—including antibodies, peptides, and natural medicines—represents the most promising direction for clinical management of radiation injuries.

Among the diverse therapeutic strategies reviewed, precision drug delivery systems based on novel materials and technologies hold the most transformative clinical potential. Nanomedicine platforms enable spatiotemporal control of drug release through integrated targeting, responsiveness, and multifunctionality. Additionally, 3D bioprinting technology opens new frontiers in tissue engineering, from assessing radiation damage with 3D‐printed fibroblast constructs to fabricating autologous cell‐based wound repair materials for accelerated healing‐all customizable bioinks permitting patient‐specific solutions. More importantly, precision drug delivery systems preserve the multitarget synergy of combination therapies while enhancing tissue specificity and therapeutic efficacy, simultaneously addressing off‐target effects and biocompatibility issues. Integrating such delivery systems with multitarget interventions—including antibodies, peptides, and natural medicines—represents the most promising direction for clinical management of radiation injuries.

Although diverse therapeutic strategies show substantial efficacy in rodent models, the bioavailability issues confronting their clinical translation constitute a critical barrier, particularly pronounced for natural compounds. Natural medicines extensively discussed herein, such as, curcumin, resveratrol, and quercetin, despite demonstrating multitarget synergistic effects and favorable safety profiles in preclinical studies, remain constrained in clinical application by compositional complexity, standardization difficulties, and incomplete mechanistic understanding. Second, the complex pathogenesis of radiation injury involves intricate crosstalk between multiple biological pathways, rendering single‐target therapeutic approaches largely ineffective. A paradigm shift from traditional “single‐target” concepts to comprehensive investigations of cellular interactions, core regulatory networks, and critical signaling pathways is urgently needed to develop more effective interventions. Notably, senescence, as an emerging therapeutic concept in clinical management, provides novel targets for radiation injury treatment. Senolytic agents (such as, navitoclax/ruxolitinib and the dasatinib/quercetin combination) selectively eliminate senescent cells and reduce SASP‐mediated injury; however, their clinical translation necessitates judicious assessment of both short‐term and long‐term adverse effects. Conversely, the clinical manifestation of radiation injury depends heavily on radiation parameters (dose, field size, and fractionation) and on multiorgan interactions, further complicating diagnosis and management. Finally, bioavailability challenges require addressing through delivery innovations including nanoparticle encapsulation, liposomal formulations, and prodrug design to enhance bioavailability, circumvent first‐pass metabolism, and improve tissue‐specific targeting.

Regarding these core challenges confronting clinical translation, future research directions should first focus on establishing translational research model systems more concordant with human pathophysiological characteristics. Targeted development of large animal models, such as, nonhuman primates or porcine species, employing fractionated radiotherapy regimens consistent with clinical practice, proves essential for accurately evaluating the efficacy and safety of therapeutic strategies. Concurrently, utilizing multiomics technologies including spatial transcriptomics and single‐cell sequencing to systematically resolve critical cell subpopulations and signaling pathways driving injury progression at various postirradiation timepoints will provide precise molecular atlases for clinical intervention. Second, leveraging big data models to optimize multitarget combination therapeutic regimens is imperative. Employing systems pharmacology and computational modeling approaches to predict drug–drug interactions and synergistic effects, whilst determining optimal dosing schedules and dose ratios through dose‐escalation studies and pharmacokinetic/pharmacodynamic modeling, represents a crucial advance. Particularly for PCD combination therapies and senolytic strategies, standardized preclinical evaluation models should be established to systematically assess the combinatorial effects and potential toxicities of different pathway inhibitors, clarifying therapeutic windows and biomarker‐guided personalized dosing regimens. Finally, multidisciplinary collaboration and standardized clinical trial design prove critical for accelerating the clinical translation of diverse therapeutic strategies. Establishing multidomain collaborative frameworks encompassing radiation oncology, cardiovascular medicine, respiratory medicine, dermatology, hematology, nanomaterial science, and bioinformatics, whilst formulating unified efficacy evaluation endpoints, biomarker validation protocols, and long‐term follow‐up strategies, ensures comparability and clinical utility of research findings. By systematically addressing these challenges, the field can achieve breakthroughs in radiation injury management, ultimately improving long‐term survival and quality of life for patients undergoing radiotherapy.

This review synthesizes current knowledge on radiation‐induced injured organs and blood, elucidating the complex interaction networks underlying disease progression and their therapeutic implications. We have critically evaluated diverse mechanistically grounded treatments, with particular emphasis on emerging biomaterial‐based approaches. It is our hope that this comprehensive analysis will stimulate more rigorous investigations to address the unmet needs in radiation injury management.

## Author Contributions

XM, XZ, and PZ contributed equally to this work and drafted the initial manuscript. ZC, ZL, YW, XH, and JY skillfully crafted the illustrations and organized the tables for the paper, enhancing its visual representation. YH and QS contributed to the compilation of some literature. ML and NL contributed to the conceptualization and critical revision of the manuscripts. All authors read and approved the final manuscript.

## Ethics Statement

The authors have nothing to report.

## Conflicts of Interest

The authors declare no conflicts of interest.

## Data Availability

The authors have nothing to report.
